# High‐performance electro‐optic materials featuring enhanced thermal stability through dual‐donor structural crosslinking engineering

**DOI:** 10.1002/smo2.70052

**Published:** 2026-05-06

**Authors:** Yu Zhang, Ziyun Zheng, Zhihan Huang, Zhifan Liu, Youling Chen, Fuyang Huo, Fenggang Liu, Xiubin Xu

**Affiliations:** ^1^ School of Chemistry and Chemical Engineering Guangzhou University Guangzhou China

**Keywords:** Diels–Alder (DA) reaction, electro‐optic coefficient, Huisgen cycloaddition reaction, long‐term stability, nonlinear optical materials

## Abstract

Organic electro‐optic (EO) materials combining high EO efficiency with robust thermal stability are essential for next‐generation optoelectronic transceivers. However, limited chromophore loading and poor thermal stability during poling processing and practical application have restricted their EO performance. Here, a dual‐donor crosslinking strategy is proposed through the synthesis of highly efficient binary crosslinkable dual‐donor chromophores (YZ1–YZ6) based on anthracene–acrylate, anthracene–maleimide and maleimide–furan Diels–Alder reactions, as well as azide–alkyne Huisgen cycloaddition. Following electric‐field poling, polymeric crosslinked networks are formed at designated temperatures, effectively locking molecular orientation and markedly enhancing thermal stability. The resulting crosslinked films exhibit large EO coefficients of 257–301 pm/V and elevated glass transition temperatures (*T*_g_) of 107–187°C, together with high chromophore densities of 3.73–4.26 × 10^20^ molecules cm^−3^. Long‐term thermal aging at 85°C demonstrates excellent stability, with 2:1 YZ1:YZ2 and 2:1 YZ5:YZ6 retaining 99.68% and 95.01% of their initial r_33_ values after 500 h, respectively. This work provides an effective molecular‐engineering strategy for the systematic development of high‐performance organic EO materials.

## Graphical abstract

A groundbreaking dual‐donor crosslinking strategy was reported to significantly enhance the thermal stability, achieving remarkable EO coefficients (257–301 pm/V), glass transition temperatures (*T*_g_) ranging from 107–187°C, and ultrahigh chromophore densities (3.73–4.26 × 10²⁰ molecules/cm³) with exceptional long‐term stability after 500 h at 85°C.

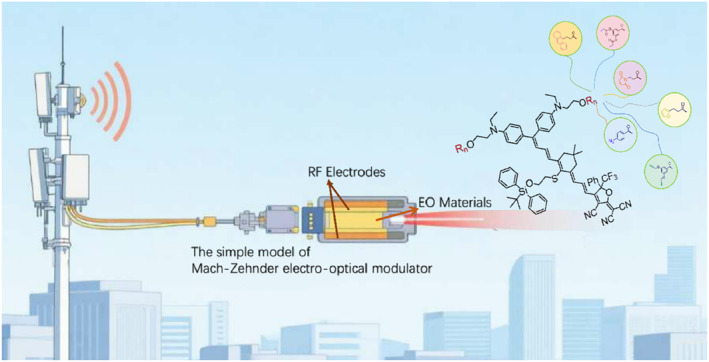

## 1 INTRODUCTION

Advanced technologies in 5G/6G communications,^[1]^ radar detection, terahertz communications,^[2]^ and electro‐optical modulators^[3]^ are driving rapid progress in photonics and related high‐tech sectors,[^4^, ^5^] As a fundamental pillar of modern communication systems, wireless networks are evolving toward single‐wavelength optical channels with line rates exceeding 400 Gb s^−1^, targeting ultra‐high‐speed signal transmission. Nevertheless, these advances remain constrained by cost, device footprint, and energy efficiency.

Inorganic materials such as lithium niobate (LiNbO_3_) have long dominated electro‐optic applications,^[6]^ enabling the fabrication of compact Mach–Zehnder modulators with modulation frequencies up to 70 GHz. However, LiNbO_3_‐based optical modulators suffer from several inherent limitations, including a relatively low electro‐optic coefficient (r_33_ ≈ 30 pm/V), which is insufficient for next‐generation high‐performance electro‐optic materials.^[7]^ In addition, these modulators are typically bulky and require traveling‐wave electrodes, complex device architectures, and high operating power.[^8^, ^9^] Consequently, the development of novel, low‐cost photonic materials that simultaneously offer high spectral efficiency and single‐channel capability is essential for next‐generation optoelectronic transceivers.

Organic electro‐optic (EO) materials have emerged as promising candidates in photonics research,[^10^, ^11^] owing to their outstanding attributes, including high electro‐optic coefficients, low dielectric constants, high optical damage thresholds, multi‐terahertz bandwidths, ultrafast response speeds, and facile processability.^[12]^ Their excellent integrability further enables co‐fabrication with semiconductor microelectronic devices, highlighting their broad potential in high‐speed information processing and optical communication. Current organic EO materials span several categories, each with distinct advantages and limitations. Guest–host doped EO systems rely on the physical incorporation of chromophores into polymer matrices.^[13]^ Although these materials are straightforward to prepare, they typically exhibit low EO coefficients due to chromophore loading limitations and reduced glass transition temperatures (*T*_g_) compared to neat polymers.[^14^, ^15^] Dendritic chromophore‐based EO materials can form free‐standing films and achieve high EO coefficients as a result of their large molecular weights[^16^, ^17^, ^18^, ^19^, ^20^]; however, their relatively low *T*_g_ restricts practical applicability. Main‐chain^[21]^ and side‐chain polymer‐based EO materials display higher *T*_g_ values,^[22]^ yet their synthesis requires substantial chromophore content, and the EO coefficients generally remain below 200 pm/V owing to chromophore loading constraints.^[23]^ Increasing chromophore loading is therefore critical for enhancing EO performance. Self‐assembled chromophores exploit π–π stacking interactions to attain high EO coefficients while maintaining long‐term poling orientation stability at room temperature.^[24]^ Nonetheless, their thermal stability at elevated temperatures remains inadequate.

The emergence of cross‐linked EO materials has effectively addressed the thermal stability issues inherent in organic EO materials at elevated temperatures.[^25^, ^26^] Early developments in crosslinked EO materials primarily focused on polymer‐host systems, wherein crosslinkable groups were introduced separately into both the polymer matrix and the chromophores.[^27^, ^28^] In such configurations, the chromophore loading is typically limited to less than 25 wt%, consequently restricting the EO coefficient (r_33_) of the cross‐linked system to values generally below 150 pm/V due to the low chromophore content.^[29]^ To overcome the limitation of low EO coefficients in polymer‐host crosslinked materials, binary cross‐linked systems were designed and synthesized by incorporating crosslinkable functionalities into two distinct chromophores.[^30^, ^31^, ^32^] Benefiting from 100 wt% chromophore loading and efficient crosslinking reactions, these binary crosslinked chromophore systems achieve an optimal balance between high EO coefficients (250–300 pm/V) and elevated glass transition temperatures (*T*_g_ ≈ 160–180°C).[^33^, ^34^, ^35^] The BAH‐X series of binary cross‐linked materials, which employ tri‐heteroatom‐modified bis(arylamino) hybrids as electron donors, initially achieved an electro‐optic (EO) coefficient (r_33_) of 420 pm/V with a glass transition temperature (*T*_g_) of 158°C via a high‐temperature cross‐linking process. Notably, when subjected to a longer cross‐linking process at a lower temperature, these materials yielded an even higher r_33_ of 670 pm/V, though the *T*_g_ decreased to 151°C^[36]^; however, this enhancement comes at the expense of a reduced *T*_g_ of approximately 150°C.

**FIGURE 1:**
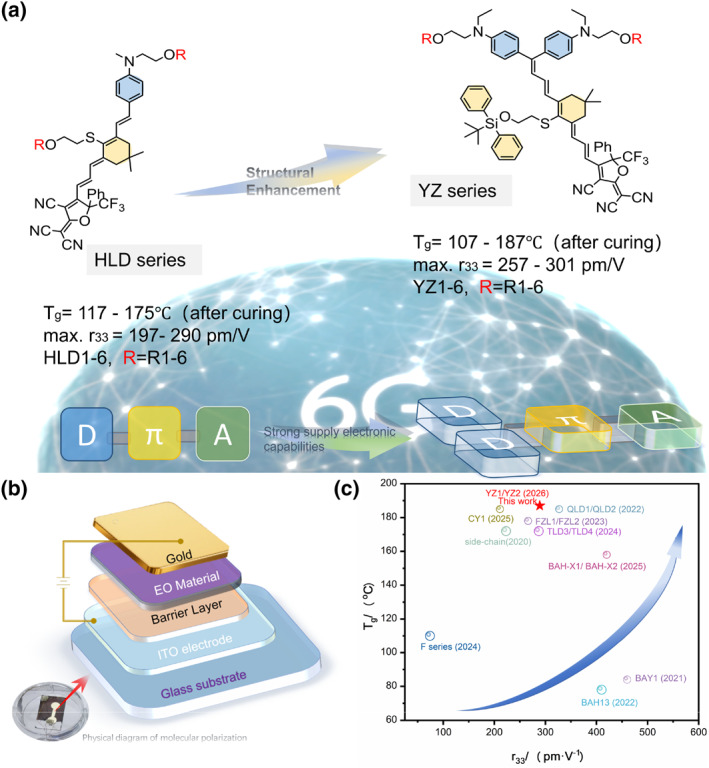
(a) Performance comparison of YZ‐series electro‐optic materials with the HLD‐series counterparts. (b) Schematic illustration and detailed architecture of the device structure. (c) Benchmarking of YZ‐series electro‐optic materials against recently reported high‐performance electro‐optic materials.[^10^, ^16^, ^17^, ^31^, ^33^, ^34^, ^35^, ^36^]

Despite these promising results, the research on binary pure chromophore crosslinked systems remains in its infancy. A significant bottleneck persists in existing material systems: the glass transition temperature (*T*_g_) struggles to surpass the critical 200°C threshold, severely limiting their potential applications in high‐temperature extreme environments. Therefore, the development of materials featuring both higher EO coefficients and higher *T*_g_ values is an urgent necessity. Addressing this, we report the first introduction of a bis(N‐ethyl‐N‐hydroxyethyl)aniline double‐donor into a binary crosslinked system, synthesizing the YZ1–YZ6 series (Figure 1). In contrast to traditional binary crosslinked materials where crosslinkable groups are typically modified on the chromophore's donor and bridge, our strategy introduces six distinct crosslinkable moieties—anthracene, acrylate, maleimide, furan, azide, and alkyne—specifically at both termini of the double donor. This dual‐donor incorporation and strategic positioning of cross‐linking groups significantly enhance both the EO coefficient and *T*_g_. Structural characterization reveals that the YZ series achieves r_33_ values of 257–301 pm/V and *T*_g_ ranges of 107–187°C. Most importantly, the cross‐linked YZ1 and YZ2 derivatives deliver an r_33_ of 289 pm/V and a *T*_g_ of 187°C; this *T*_g_ of 187°C represents the highest value reported to date for any binary crosslinked material.[^33^, ^34^, ^35^]

## 2 EXPERIMENTAL

### 2.1 Synthesis of chromophores

**FIGURE 2:**
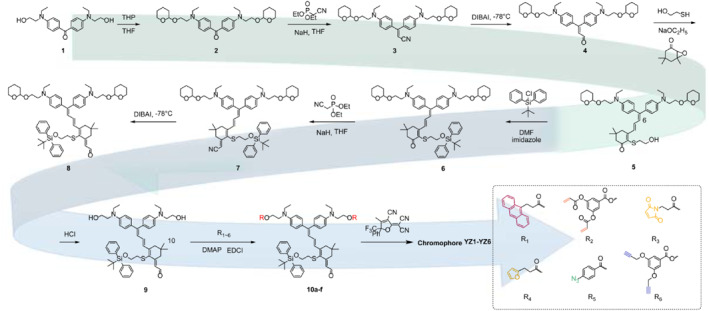
Schematic overview of the synthetic routes toward the chromophore molecules YZ1–YZ6.

The chromophores YZ1–6 feature a double‐donor architecture and were functionalized by introducing two distinct crosslinkable groups at the donor moiety. The synthetic routes for the chromophores YZ1–6 are illustrated in Figure 2. Following a 10‐step synthetic sequence, the target chromophores YZ1–6 were obtained as blue–green solids. During the synthesis, protection of the reactive hydroxyl groups was required to ensure selective functionalization.

In the first step, compound 1 was reacted with tetrahydropyran (THP) in tetrahydrofuran (THF) to protect the two hydroxyl groups on the donor unit, yielding compound 2. Compound 3 was subsequently prepared via a Wittig–Horner reaction between compound 2 and diethyl (cyanomethyl)phosphonate catalyzed by sodium hydride. Reduction of the nitrile group in compound 3 with diisobutylaluminum hydride (DIBAL) at low temperature afforded aldehyde 4. Compound 5 was then synthesized through a Knoevenagel condensation between aldehyde 4 and isophorone. Protection of the mono‐hydroxyl group on the π‐conjugated bridge using tert‐butyldimethylsilyl chloride (TBDMS) yielded compound 6. The subsequent reaction with diethyl (cyanomethyl)phosphonate in the presence of sodium hydride produced trienenenitrile 7. Reduction of the nitrile functionality in compound 7 with DIBAL afforded aldehyde 8. Acidic deprotection of the TBDMS group in compound 8 yielded compound 9 bearing two hydroxyl groups.

Through Steglich esterification, the dihydroxyl groups of compound 9 were functionalized with anthracenyl, benzyloxy, maleimido, furanyl, azido, and alkynyl moieties to afford intermediates 10a–10f. Finally, condensation of aldehydes 10a–10f with the CF_3_–TCF acceptor yielded the target blue–green solid chromophores YZ1–6.

## 3 RESULTS AND DISCUSSION

### 3.1 Thermal stability

The thermal properties of the chromophores YZ1–6 were investigated by thermogravimetric analysis (TGA) to determine the decomposition temperature (*T*_d_) and differential scanning calorimetry (DSC) to measure the glass transition temperature (*T*_g_), both conducted under a nitrogen atmosphere (Figure 3 and Figures S14–S27). As shown in Figure 3a, all six chromophores exhibit *T*_d_ values above 200°C, indicating good intrinsic thermal stability.

**FIGURE 3:**
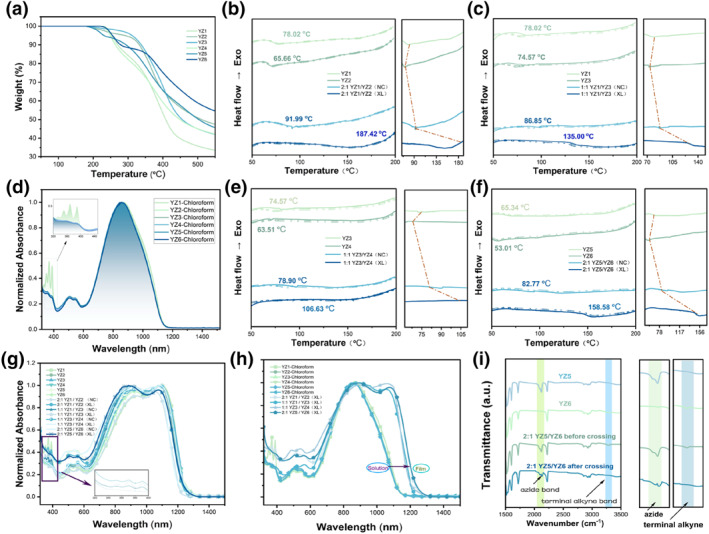
(a) Thermogravimetric analysis (TGA) curves of chromophores YZ1–YZ6. (b, c, e, f) Differential scanning calorimetry (DSC) curves of binary chromophore systems YZ1/YZ2, YZ1/YZ3, YZ3/YZ4, and YZ5/YZ6, respectively. (d) UV–vis absorption spectra of YZ1–YZ6 in chloroform. (g) UV–vis absorption spectra of YZ1–YZ6 thin films before and after crosslinking. (h) Comparison of UV–vis absorption spectra of YZ1–YZ6 in solution and solid states. (i) Fourier‐transform infrared (FTIR) spectra of the YZ5/YZ6 system before and after crosslinking.

The glass transition temperature plays a critical role in determining the poling temperature and long‐term orientational stability of chromophore‐based materials and thus strongly influences the stability of the electro‐optic coefficient. When the operating temperature exceeds *T*_g_, the poled chromophore molecules tend to relax toward an isotropic orientation, leading to a pronounced reduction in electro‐optic performance. As shown in Figure 3b,c,e,f, among the six chromophores, YZ1 exhibits the highest *T*_g_ (78.02°C), while YZ6 shows the lowest *T*_g_ (53.01°C). These values are comparable to those reported for chromophores employing CF_3_–TCF acceptors. The observed variations in *T*_g_ among YZ1–6 are primarily attributed to differences in the rigidity of the functional groups attached to the donor moiety.

Binary blending of chromophores significantly enhances *T*_g_ compared to that of the individual components. Specifically, 2:1 YZ1/YZ2, 1:1 YZ1/YZ3, 1:1 YZ3/YZ4, and 2:1 YZ5/YZ6 mixtures exhibit increased *T*_g_ values ranging from 78.9°C to 91.99°C. During DSC heating, partial crosslinking can occur; however, the extent of crosslinking remains limited due to the short heating duration, resulting in only moderate *T*_g_ enhancement.

To further evaluate the effect of complete crosslinking, the *T*_g_ values of fully crosslinked chromophore systems were examined. Anthracene–acrylate, anthracene–maleimide, and maleimide–furan Diels–Alder (DA) reactions, as well as azide–alkyne Huisgen cycloaddition reactions, were induced during thermal treatment under optimized conditions (YZ1/YZ2: 160°C for 60 min; YZ1/YZ3: 135°C for 30 min; YZ3/YZ4 and YZ5/YZ6: 150°C for 60 min). Under these conditions, the crosslinking reactions proceeded to a high extent, converting the initially small‐molecule chromophores into polymeric networks. As a result, the *T*_g_ values of the 2:1 YZ1/YZ2, 1:1 YZ1/YZ3, 1:1 YZ3/YZ4, and 2:1 YZ5/YZ6 systems increased substantially to the range of 106.63–187.42°C, with the highest *T*_g_ observed for the crosslinked 2:1 YZ1/YZ2 system.

Two main factors govern the *T*_g_ of the chromophores after crosslinking. The first is the nature of the crosslinking reaction. Notably, the *T*_g_ of YZ3/YZ4 (107°C) is markedly lower than those of other crosslinked systems (135–187°C), which may be attributed to the relatively low retro‐DA temperature of the maleimide–furan DA reaction and fewer crosslinkable groups. The second factor is the number of crosslinkable groups; a higher density of reactive groups increases the probability of network formation. Consequently, the *T*_g_ of YZ1/YZ3 (135°C) is lower than that of YZ1/YZ2 (187°C), reflecting the lower number of reactive sites in the maleimide‐functionalized YZ3 compared to the acrylate‐functionalized YZ2. Increasing the maleimide group density within the chromophore structure may therefore further enhance *T*_g_. Moreover, the anthracene–maleimide reaction offers advantages such as faster kinetics and milder reaction conditions relative to the anthracene–acrylate system, potentially contributing to improved chromophore stability during crosslinking. The high *T*_g_ observed for YZ5/YZ6 (158°C) further demonstrates that azide–alkyne‐based Huisgen cycloaddition is well suited for binary crosslinked neat chromophore systems.[^31^, ^32^]

### 3.2 Optical properties

To evaluate the charge‐transfer characteristics of the chromophores YZ1–6 bearing different functional groups on the donor and π‐bridge units, their UV–vis absorption spectra were measured in various solvents as well as in thin films before and after crosslinking (Table 1). In chloroform, the maximum absorption wavelengths (λ_max_) of YZ1–6 span from 846 to 871 nm, indicating that the introduction of crosslinkable groups does not perturb the primary conjugated backbone. Compared with structurally related chromophores (e.g., HLD) incorporating conventional aniline donors and CF_3_–TCF acceptors,^[30]^ YZ1–6 exhibited a pronounced red shift, suggesting enhanced molecular hyperpolarizability.

**TABLE 1:** Optical and Thermal property data of the chromophores.

Cmpd	*T*_d_ (°C)	λ_max_^a^	λ_max_^b^	Δλ^c^	λ_max_^d^	*T*_g_^e^ (°C)	*T*_g_^f^ (°C)	*T*_g_^g^ (°C)
YZ1	253.53	871	835	26	1094	78.02	91.99^h^	187.42^h^
YZ2	290.66	846	831	15	1082	65.66		
YZ3	311.36	859	815	44	1092	74.57	86.85^i^	135.00^i^
YZ4	249.37	857	813	44	1086	63.51	78.90^j^	106.63^j^
YZ5	230.79	856	811	45	1090	65.34	82.77^k^	158.58^k^
YZ6	255.23	858	814	44	1086	53.01		

*Note*: ^a,b,d^ (nm) was measured in chloroform, dioxane and in film, respectively. ^c^(nm) was the difference between λ_max_^a^ and λ_max_^b^. ^e^Individual chromophores. ^f^ binary blend, ^g^after crosslinking. ^h^2:1YZ1:YZ2. ^i^1:1 YZ1:YZ3. ^j^1:1 YZ3:YZ4. ^k^2:1 YZ5:YZ6.

The UV–vis–NIR absorption spectra of the six chromophores were further investigated in a series of aprotic solvents with varying polarity to assess their solvatochromic behavior over a broad dielectric range (Figure 3d and Figure S28). The most significant bathochromic shift in λ_max_ occurs when transitioning from 1,4‐dioxane to dichloromethane, whereas further increases in solvent polarity lead to spectral saturation in highly polar solvents such as acetone and acetonitrile. Notably, the YZ chromophores initially display a bathochromic shift followed by a hypsochromic shift in more polar environments, a phenomenon known as inverted solvatochromism.^[37]^ This behavior suggests that the YZ chromophores approach or even exceed the cyanine limit, entering a zwitterionic electronic regime in highly polar media. Similar solvent‐dependent spectral trends have been reported for certain merocyanine dyes as well as tetrahydroquinolinyl or heteroaryl‐amino‐based tetraene chromophores.^[38]^ The inverted solvatochromism observed for YZ chromophores can be attributed to their proximity to the cyanine limit. The strong push‐pull character, derived from the dual donors and the CF_3_‐TCF acceptor, creates a highly zwitterionic ground state. In polar solvents, this ground state is preferentially stabilized relative to the excited state, resulting in a blue shift of the absorption maximum.

The progress of the crosslinking reactions was monitored by UV–vis absorption spectroscopy of the thin films. Measurements were performed on individual chromophores YZ1–6, their binary mixtures (2:1 YZ1/YZ2, 1:1 YZ1/YZ3, 1:1 YZ3/YZ4, and 2:1 YZ5/YZ6), and the corresponding crosslinked films (Figure 3g). After thermal curing, a pronounced decrease in the characteristic anthracene absorption bands at approximately 350, 370, and 390 nm was observed (Figure 3g), confirming efficient crosslinking between anthracene and acrylate groups in the YZ1/YZ2 system. A similar reduction in anthracenyl absorption is evident for the YZ1/YZ3 system, consistent with the occurrence of a Diels–Alder cycloaddition between the anthracenyl and maleimide groups at 135°C. In contrast, the azide (YZ5) and alkynyl (YZ6) functionalities do not exhibit distinct UV absorption features, rendering direct spectroscopic monitoring of crosslinking less straightforward. Nevertheless, a noticeable blue shift in the absorption maximum of the 2:1 YZ5/YZ6 blend after curing—analogous to that observed for YZ1–4—provides indirect evidence of successful crosslinking.

The Huisgen 1,3‐dipolar cycloaddition reaction between YZ5 and YZ6 was further examined by tracking changes in Fourier‐transform infrared (FTIR) spectra during thermal treatment at 150°C for 60 min. In particular, the asymmetric stretching vibration of the azide group at approximately 2102 cm^−1^ and the characteristic terminal alkyne stretching band near 3300 cm^−1^ were monitored. As shown in Figure 3i, these diagnostic peaks decrease by approximately 70% after curing, confirming the high efficiency of the cycloaddition reaction. In addition, the curing process converts the initially highly soluble YZ1/YZ2, YZ1/YZ3, and YZ5/YZ6 blends into mechanically robust films with excellent solvent resistance capable of withstanding exposure to polar solvents such as tetrahydrofuran (THF), trichloroethane (TCE), and acetone. Collectively, these results demonstrate the successful implementation of both Huisgen 1,3‐dipolar cycloaddition and Diels–Alder crosslinking reactions involving anthracenyl–acrylate, anthracenyl–maleimide, and azide–alkyne functional pairs.

### 3.3 Theoretical calculations

Density functional theory (DFT) calculations were employed to optimize the molecular geometries, enabling determination of the most stable atomic configurations. The first‐order hyperpolarizability (*β*), which directly governs electro‐optic performance, is a key parameter in the rational design of high‐performance electro‐optic materials. In addition, analysis of the highest occupied molecular orbital and lowest unoccupied molecular orbital energy levels, together with the corresponding energy gap (Δ*E*), provides further insight into the optical properties and intramolecular charge‐transfer characteristics of the chromophores. The DFT‐derived results therefore constitute an essential basis for evaluating the chromophore performance and facilitate a deeper understanding of the underlying structure–property relationships.

To compare the molecular geometries, microscopic nonlinear optical responses, and intramolecular charge‐transfer characteristics of different chromophores, theoretical calculations were performed for YZ1–6 using Gaussian 09 software. Density functional theory (DFT) calculations were carried out with the CAM‐B3LYP functional in combination with the 6‐31G (d, p) basis set.[^39^, ^40^] In these calculations, all molecules were assumed to adopt all‐trans conformations to obtain optimized chromophore geometries. The calculated microscopic first‐order hyperpolarizabilities (*β*), dipole moments (*μ*), electron density distributions, and energy level differences between the ground and excited states are summarized in Table 2.

**TABLE 2:** Summary of UV−Vis and DFT data.

Cmpd	λ_max_ (nm)	△E (DFT)^a^ (eV)	μ^b^ (D)	Vacuum^c^ (10^−30^esu)	Tetrahydrofuran (10^−30^esu)	Chloroform (10^−30^esu)	Toluene (10^−30^esu)
YZ1	871	1.81	30.50	1284	6312	5429	3945
YZ2	846	1.83	32.89	1387	6149	5581	3130
YZ3	859	1.82	29.21	1367	6924	5714	3727
YZ4	857	1.81	25.83	1233	6565	5425	3497
YZ5	856	1.79	27.72	1194	6873	5723	3806
YZ6	858	1.77	27.62	1328	6683	5478	3605

^a^ Was calculated from DFT calculations.

^b^ Is the total dipole moment.

^c^ Is the first‐order hyperpolarizability calculated from DFT calculations in vacuum.

**FIGURE 4:**
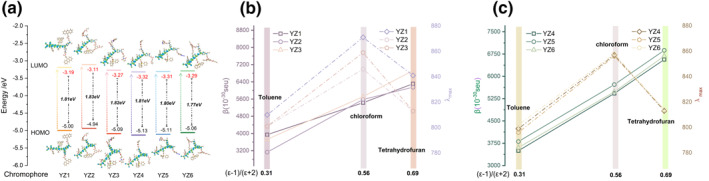
(a) HOMO–LUMO energy gaps (Δ*E*) of chromophores YZ1–YZ6. (b, c) Solvatochromic dependence of the calculated *β* values and the absorption maximum (λ_max_) for chromophores YZ1–YZ3 and YZ4–YZ6, respectively.

As shown in Figure 4a and Table 2, the HOMO–LUMO energy gaps (Δ*E*) of chromophores YZ1–6 fall within a narrow range of 1.73–1.82 eV. The similarity of Δ*E* values among the six chromophores indicates that the introduction of crosslinkable functional groups does not perturb the primary conjugated backbone. Consistent with this observation, all six chromophores exhibit comparable first‐order hyperpolarizabilities in vacuum, which can be attributed to their nearly identical conjugated structures.

The first‐order hyperpolarizabilities of the chromophores were further calculated in three different solvents—chloroform, tetrahydrofuran, and toluene—using the M06‐2X/6‐31+G(d) level of theory within an implicit solvent (PCM) framework to elucidate solvent effects on hyperpolarizability and dipole moment (Figure 4b,c and Table 2). Among the three solvents examined, chromophores YZ1–6 exhibit their highest first‐order hyperpolarizabilities in tetrahydrofuran, with YZ3 showing the largest value (6924 × 10^−30^ esu) and YZ2 the smallest (6149 × 10^−30^ esu), consistent with the trends obtained from vacuum calculations. Overall, tetrahydrofuran yields the largest *β* values for all chromophores, followed by chloroform, while toluene results in the lowest values.

As shown in Figure 4b,c, the solvent dependence of the first‐order hyperpolarizability differs slightly from that of the maximum UV–vis absorption wavelength (λ_max_). In contrast to the hyperpolarizability trend, the absorption maxima of these chromophores occur in chloroform.

### 3.4 Electric field induced polarization

Electro‐optic (EO) materials were dissolved in trichloroethane (TCE) at concentrations of 8–10 wt% and sonicated for 15 min to ensure complete dissolution. The resulting solutions were filtered through a 0.2 μm PTFE membrane and spin‐coated onto ITO/glass substrates either with or without benzocyclobutene (BCB) barrier layers.^[41]^ Spin coating was performed using a three‐step protocol: an initial spreading step at 500 rpm for 5 s, a deposition step at 850 rpm for 30 s, followed by a thinning step at 1500 rpm for 30 s. The coated films were subsequently dried either at room temperature in a vacuum oven overnight or at 65°C for a defined period (typically 6–12 h). Optical profilometry confirmed EO film thicknesses in the range of 1–2 μm. For device fabrication, patterned gold electrodes were deposited onto the films by sputtering through a shadow mask.

**FIGURE 5:**
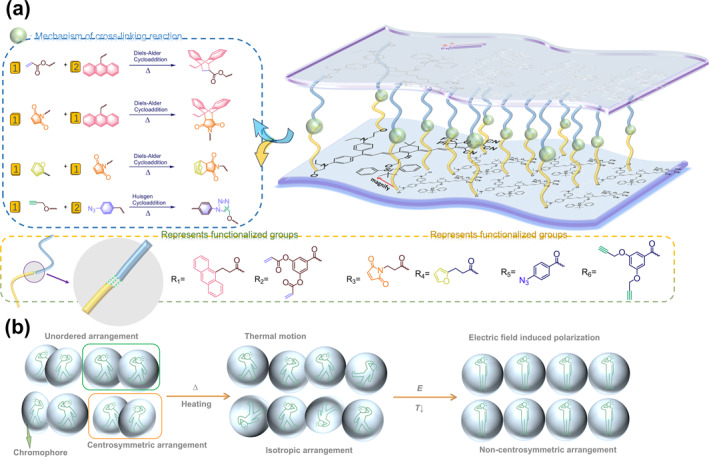
(a) Schematic illustration of the crosslinking process for chromophores YZ1–YZ6. (b) Molecular polarization schematic.

For non‐crosslinked EO thin films, electric‐field poling was conducted by applying an external electric field at room temperature, heating the sample to its glass transition temperature (*T*_g_), maintaining this temperature for approximately 10 min to allow chromophore alignment, and subsequently cooling to ambient temperature prior to field removal, as illustrated in Figure 5. Throughout the poling process, current, voltage, temperature, and relative r_33_ values were monitored to optimize both poling and crosslinking conditions. After completion of poling and cooling to near room temperature, the r_33_ values of the poled films were measured using the Teng–Man method on a custom‐built setup operating at 1310 nm as listed in Table 3.^[42]^ Standard error analysis for r_33_ and poling efficiency was performed according to established procedures. To mitigate film cracking during high‐temperature poling and crosslinking, a stepwise poling strategy was implemented, adapted from previously reported chromophore crosslinking methodologies.^[43]^

**TABLE 3:** Electric field poling data for EO chromophores in bulk devices.

Chromophore	ρ_N‐ave_ (×10^20^ molecules cm^−3^)^a^	Poling temp (°C)	r_33_/E_p_ [nm^2^/V^2^]^b^/	r_33/_(E_p_ρ_N_)^c^	Max. r_33_ (pm·V^−1^)
YZ1	3.75	83	2.92 ± 0.10	0.78 ± 0.03	246
YZ2	3.70	71	2.81 ± 0.09	0.76 ± 0.02	235
2:1 YZ1:YZ2 (uncrosslinked)	3.73	97	3.34 ± 0.11	0.90 ± 0.02	337
2:1 YZ1:YZ2 (crosslinked)	3.73	110–160	2.37 ± 0.09	0.64 ± 0.02	289
YZ3	4.17	80	3.00 ± 0.10	0.72 ± 0.02	271
1:1 YZ1:YZ3 (uncrosslinked)	3.95	92	3.48 ± 0.10	0.88 ± 0.02	359
1:1 YZ1:YZ3 (crosslinked)	3.95	100–135	2.43 ± 0.08	0.62 ± 0.02	298
YZ4	4.35	69	3.09 ± 0.09	0.71 ± 0.02	281
1:1 YZ3:YZ4 (uncrosslinked)	4.26	84	3.41 ± 0.12	0.80 ± 0.03	353
1:1 YZ3:YZ4 (crosslinked)	4.26	100–135	2.11 ± 0.10	0.50 ± 0.02	257
YZ5	4.21	70	3.13 ± 0.11	0.74 ± 0.02	273
YZ6	3.95	58	2.95 ± 0.11	0.75 ± 0.02	259
2:1 YZ5:YZ6 (uncrosslinked)	4.10	88	3.45 ± 0.11	0.84 ± 0.03	360
2:1 YZ5:YZ6 (crosslinked)	4.10	100–150	2.46 ± 0.08	0.60 ± 0.02	301

^a^ Number density (assumes mass density of 1 g/cm^3^).

^b^ Average from multiple poling experiments.

^c^ Poling efficiency per number density (nm^2^/V^2^/(10^20^molecules/cm^3^)).

### 3.5 Testing of electro‐optical coefficients

**FIGURE 6:**
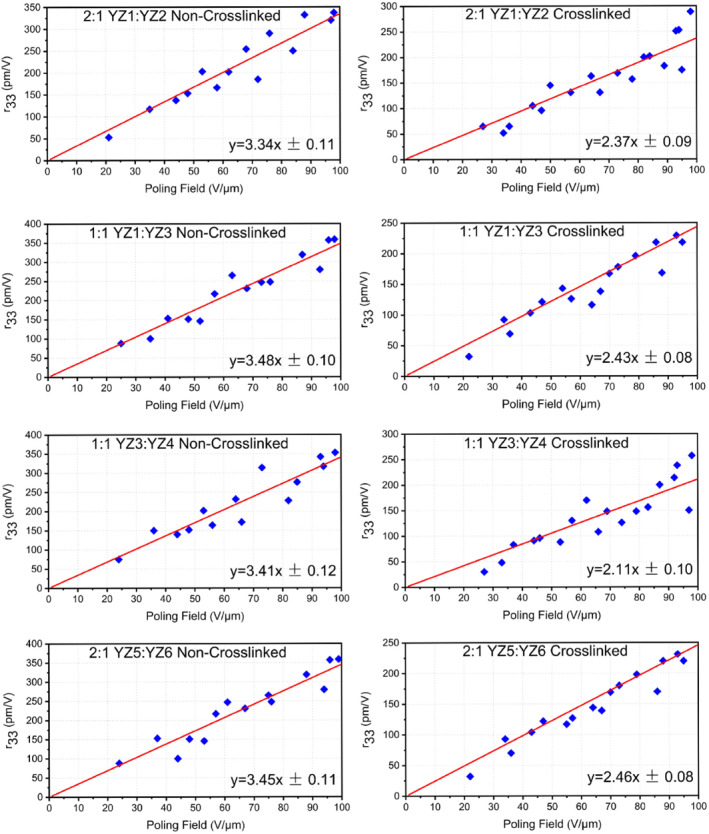
Polarization‐field dependence of the electro‐optic coefficient (r_33_) for chromophore systems YZ1/YZ2, YZ1/YZ3, YZ3/YZ4, and YZ5/YZ6 before and after crosslinking.

The electro‐optic (EO) performance of the individual chromophores was first evaluated using a conventional electric‐field poling process conducted at temperatures 5–10°C above their respective glass transition temperatures (*T*_g_). The measured maximum r_33_ values for the chromophores YZ1–YZ6 ranged from 235 to 281 pm/V. The electro‐optic (EO) coefficient of chromophores is positively correlated with their first‐order hyperpolarizability (*β*), number density (N), and poling efficiency (f). Notably, poling efficiency is predominantly governed by steric effects arising from bulky groups. Since the YZ1–YZ6 chromophores share an identical conjugated backbone, DFT theoretical calculations indicate that their first‐order hyperpolarizabilities are comparable. Consequently, the observed variations in EO coefficients primarily stem from differences in chromophore number density and the steric hindrance imposed by the diverse cross‐linkable moieties. Specifically, the superior EO coefficient exhibited by YZ4 is likely attributed to its maximized chromophore number density. As illustrated in Figure 6, Figure S29 and Table 3, the average poling efficiencies (r_33_/E_p_) for YZ1–YZ6 fall within the range of 2.81 ± 0.09–3.13 ± 0.11 nm^2^ V^−2^. These values exceed those of the analogous HLD chromophore employing an aniline donor, which can be ascribed to the stronger electron‐donating ability of the double‐donor architecture leading to enhanced *β* values.

To further assess the EO coefficients and poling efficiencies of the chromophores YZ1–YZ6 before and after crosslinking, mixtures of crosslinkable chromophores were prepared. To ensure equivalent numbers of reactive crosslinking groups, chromophore pairs YZ1/YZ2 and YZ5/YZ6 were blended at 2:1 M ratios, whereas YZ1/YZ3 and YZ3/YZ4 were mixed at 1:1 M ratios. Below a poling temperature of 110°C, the EO films behaved as conventional binary chromophore blends, which significantly enhanced the EO coefficients. As shown in Figure 6, Figure S29 and Table 3, the maximum r_33_ values of the 2:1 YZ1/YZ2, 1:1 YZ1/YZ3, 1:1 YZ3/YZ4, and 2:1 YZ5/YZ6 systems ranged from 337 to 360 pm/V, demonstrating the effectiveness of binary blending in boosting the EO performance.

Poling of crosslinked chromophore systems requires elevated temperatures (135–160°C). Prior to crosslinking, the chromophore films were vacuum‐dried at 65°C for 12 h to facilitate the application of higher electric fields. To suppress film cracking, a stepwise poling protocol was employed. Using the YZ1/YZ2 system as an example, under a constant electric field, the temperature was increased sequentially at 10°C min^−1^ to 130°C (held for 10 min), then to 140°C (held for 10 min), and finally to 150°C (held for 10 min), followed by cooling. The combined effects of crosslinking and the benzocyclobutene (BCB) charge‐injection barrier layer with a thickness of ∼100 nm enabled the poling fields to be increased to approximately 100 V μm^−1^. After crosslinking, the measured r_33_ values were 289 pm/V for 2:1 YZ1/YZ2, 298 pm/V for 1:1 YZ1/YZ3, 257 pm/V for 1:1 YZ3/YZ4, and 301 pm/V for 2:1 YZ5/YZ6. These values represent the highest reported EO coefficients for crosslinked organic electro‐optic materials to date.

The reduction in r_33_ values compared to those of the uncrosslinked binary blends indicates that crosslinking partially hinders chromophore alignment, thereby reducing poling efficiency. In addition, the nature of the crosslinkable groups and the associated reactions plays a critical role in determining the EO performance. For example, the relatively lower thermal stability of furan groups and the lower retro‐Diels–Alder temperature contribute to a reduced EO coefficient (254 pm/V). These results highlight the importance of optimizing the interplay among glass transition temperature, poling temperature, and crosslinking temperature to maximize EO coefficients. The high post‐crosslinking r_33_ values approaching 300 pm/V for the 2:1 YZ1/YZ2, 1:1 YZ1/YZ3, and 2:1 YZ5/YZ6 systems confirm the suitability of these thermally initiated reactions for high‐performance binary crosslinked chromophore systems.

### 3.6 Long‐term alignment stability test

**FIGURE 7:**
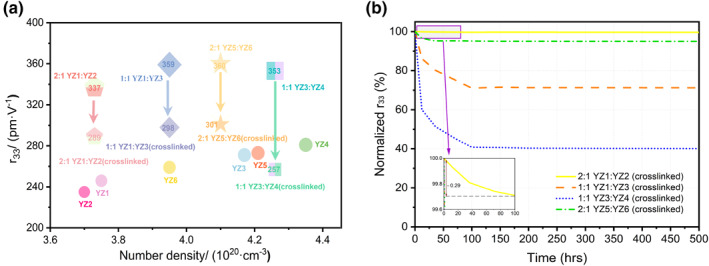
(a) Electro‐optic coefficient (r_33_) of chromophores YZ1–YZ6 and their binary mixtures as a function of number density (N). (b) Temporal stability of poled films based on YZ1/YZ2, YZ1/YZ3, YZ3/YZ4, and YZ5/YZ6 measured at 85°C under nitrogen atmosphere.

For practical commercialization, long‐term performance stability is a critical requirement. High‐temperature alignment stability was therefore evaluated as shown in Figure 7b. After thermal aging at 85°C for 500 h under a nitrogen atmosphere, the poled and crosslinked electro‐optic films of 2:1 YZ1/YZ2 and 2:1 YZ5/YZ6 retained 99.68% and 95.01% of their initial electro‐optic coefficients, respectively. In contrast, the 1:1 YZ1/YZ3 and 1:1 YZ3/YZ4 systems retained only 71.20% and 40.11% of their initial electro‐optic coefficients, respectively. These results clearly demonstrate that the elevated glass transition temperatures achieved in the binary crosslinked systems play a decisive role in maintaining long‐term chromophore alignment stability.

## 4 CONCLUSION

In summary, we have successfully addressed the critical trade‐off between electro‐optic activity and thermal stability in organic EO materials by engineering a novel series of binary crosslinked chromophores (YZ1–YZ6). Distinct from conventional strategies that modify crosslinkable groups on the donor or bridge individually, our work pioneers the integration of a bis (N‐ethyl‐N‐hydroxyethyl)aniline double‐donor scaffold functionalized with six diverse cross‐linkable moieties (anthracene, acrylate, maleimide, furan, azide, and alkyne). This dual‐donor architecture not only maximizes chromophore loading to 100 wt% but also creates a highly rigid, densely crosslinked network upon curing.

The resulting crosslinked films demonstrate substantial electro‐optic (EO) coefficients ranging from 257 to 301 pm/V and elevated glass transition temperatures (*T*_g_) between 107 and 187°C. Notably, the film 2:1 YZ1:YZ2 stoichiometry exhibits exceptional performance, culminating in an optimized EO coefficient (r_33_) of 289 pm/V alongside a remarkably high *T*_g_ of 187°C. This *T*_g_ represents the highest value reported to date for any binary cross‐linked EO system, effectively surpassing the long‐standing ∼150–160°C ceiling observed in state‐of‐the‐art analogs (e.g., the BAH‐X series). Beyond these static metrics, the engineered networks demonstrate remarkable long‐term operational stability. In accelerated thermal aging tests at 85°C for 500 h, the optimal formulations retained 99.68% (for the 2:1 YZ1:YZ2 blend) and 95.01% (for the 2:1 YZ5:YZ6 blend) of their initial r_33_ values. This near‐perfect retention confirms that our strategic placement of multiple cross‐linking sites effectively locks molecular orientation, solving the chronic issue of poling relaxation that has limited the practical deployment of organic EO materials.

These findings establish the YZ series as a premier candidate for next‐generation photonic applications, particularly in 5G/6G communications, radar detection, and hybrid silicon‐organic modulators where operation under extreme thermal conditions and long device lifetimes are requisite. While recent advances by other groups have validated the potential of tri‐heteroatom‐modified bis(arylamino) hybrids‐donor and Diels‐Alder cross‐linking chemistries, our work pushes the boundaries of both thermal resilience and temporal stability further, providing a robust material platform for high‐temperature poling and reliable long‐term service. Future research will focus on the fabrication of low‐loss slot‐waveguide modulators based on these YZ derivatives and exploring their integration into heterogeneous photonic circuits to realize ultra‐high‐speed, energy‐efficient optical transceivers for future wireless networks.

## CONFLICT OF INTEREST STATEMENT

The authors declare no conflicts of interest.

## ETHICS STATEMENT

No animal or human experiments were involved in this study.

## Supporting information

Supporting Information S1

## Data Availability

The data that support the findings of this study are available from the corresponding author upon reasonable request.
