# Supplementary material for: High‐performance electro‐optic materials featuring enhanced thermal stability through dual‐donor structural crosslinking engineering
Source: Smart Mol. 2026 May 6;4(3):e70052. doi: 10.1002/smo2.70052 (PMC13399531; doi:10.1002/smo2.70052)
Supplement: Supplementary file 1 [file SMO2-4-e70052-s001.docx]

Supporting Information

High-Performance Electro-Optic Materials Featuring Enhanced Thermal Stability through Dual-Donor Structural Crosslinking Engineering

Yu Zhang#, Ziyun Zheng#, Zhihan Huang, Zhifan Liu, Youling Chen, Fuyang Huo, Fenggang Liu* and Xiubin Xu*

School of Chemistry and Chemical Engineering, Guangzhou University, Guangzhou 510006, P. R. China

*Corresponding authors.

E-mail: liufg6@gzhu.edu.cn, xuxb@gzhu.edu.cn.

**Table of Contents**

[1. Materials and instruments	1](#_Toc11301)

[2. Experimental	1](#_Toc27634)

[3. NMR pictures	10](#_Toc17873)

[4. Differential Scanning Calorimetry testing	1](#_Toc26516)7

[5. UV-Vis Absorption Spectroscopy	2](#_Toc17048)4

[6.Electro-optical performance test	2](#_Toc17747)5

[7 Properties of the state-of-the-art organic EO materials	2](#_Toc17340)6

[8. Comparison of different systems	2](#_Toc12005)7

[9. Reference	2](#_Toc2987)8

# 1. Materials and instruments

The chemicals used in this paper were commercially available and do not require further purification unless otherwise stated. The solvents used in the experiment like tetrahydrofuran (THF), *N,N*-dimethylformamide (DMF) and dichloromethane (DCM) were commercial ultra-dry reagents. Thin-layer chromatography on 0.25 mm-thick pre-coated silica gel plates and showed spots under UV light. Kieselgel (60-100 mesh and 200-300 mesh) silica gel chromatography was used.

The specific synthesis steps of chromophores YZ1-6 and its intermediates and the characterization data of mass spectrum, hydrogen spectrum and carbon spectrum are shown in the supporting information. ^1^H-NMR and ^13^C-NMR spectra were obtained by an Advance Bruker 500M (500 MHz) NMR spectrometer (tetramethyl silane was used as an internal reference). Mass spectra were obtained on a MALDITOF (matrix-assisted laser desorption/flight ionization). BIFLEX III (Broker Inc.) spectrometer. UV-Vis spectra were performed on a Cary 5000 spectrometer.Infrared spectroscopy was performed on a Nicolet lS50 spectrometer. TGA was determined by TA5000-2950TGA (TA co) with a heating rate of 10 C min^-1,^ under nitrogen protection. Glass-transition temperature (T_g_) was measured by differential scanning calorimetry (DSC) with a heating rate of 10 ℃ min^−1^ under the protection of nitrogen.

# 2. Experimental

**2.1 Synthesis and characterization of chromophore**

The synthesis route of chromophore YZ1-6 is shown in Figure S1.The synthesis innovation of chromophore YZ1-6 mainly lies in the use of a donor 4,4'-bis(diethylamino)benzophenone with high electro-optical properties, the derivatized chain on the electron bridge is replaced by a large silane containing phenyl to enhance the overall rigidity, and then the CF_3_-TCF acceptor with better comprehensive performance is used to observe the overall structural performance. In the synthesis step, a stepwise protection approach is used to protect the hydroxyl groups on the donor and the hydroxyl groups on the electron bridge, respectively, to avoid yield and structural effects.

Specific synthetic steps for the chromophores YZ 1-6 and their intermediates, including characterization data for mass, hydrogen, and carbon spectra, are described in the Supplementary Information below. The ^1^H-NMR and ^13^C-NMR spectra were obtained by an Advance Bruker 500M ( 500 MHz ) nuclear magnetic resonance spectrometer ( with tetramethylsilane as the internal standard ). The chromophore mass spectrum was obtained using a BIFLEX III ( Broker Inc. ) mass spectrometer, the infrared spectrum was obtained using a Nicolet lS50 spectrometer, and the ultraviolet-visible spectrum was performed on a Cary 5000 spectrometer. TGA was measured by TA5000-2950TGA ( TA co ) at a heating rate of 10 °C·min^-1^ in nitrogen atmosphere. The glass transition temperature (T_g_) was determined by differential scanning calorimetry (DSC), under nitrogen protection, maintaining a heating rate of 10 °C·min^-1^.

**Figure S1.** Synthesis route for Chromophores YZ1-6.

**2.2 Synthesis of intermediates and push-pull chromophores**

**2.2.1 Synthesis reference for compound 1^[1]^**

**2.2.2 Synthesis of compound 2**

Compound 1 (12.74 g, 35.74 mmol) was dissolved in tetrahydrofuran (40 ml) with tetrahydropyran (6.16 g, 71.48 mmol) under argon protection for 3 hours at room temperature. The solvent is removed by a rotary evaporator and the crude product is purified by column chromatography (in which the eluent petroleum ether:ethyl acetate (10:1 gradient to 3:1)) is purified. The yield of 80% light yellow oily product 2 (15.00 g, 28.59 mmol) was obtained. MS (MALDI) (M+, C_31_H_44_N_2_O_5_): calcd: 524.33; found :524.39. ^1^H NMR (500 MHz, Chloroform-*d*) δ 7.76 – 7.71 (m, 4H, ArH), 6.70 (d, *J* = 8.5 Hz, 4H, ArH), 4.60 (t, *J* = 4.4, 2.9 Hz, 2H, OCH), 3.95 – 3.79 (m, 4H, OCH_2_), 3.65 – 3.61 (m, 4H, OCH_2_), 3.61 – 3.51 (m, 4H, NCH_2_), 3.50 – 3.47 (m, 4H, NCH_2_), 1.85 – 1.67 (m, 4H, CH_2_), 1.61 – 1.54 (m, 4H, CH_2_), 1.51 (t, *J* = 6.1 Hz, 4H, CH_2_), 1.21 (t, *J* = 7.0 Hz, 6H, CH_3_). ^13^C NMR (126 MHz, Chloroform-*d*) δ 193.70, 150.60, 132.50, 126.03, 110.37, 99.27, 65.03, 62.41, 50.23, 45.68, 30.70, 25.48, 19.54, 12.26, 1.13.

**2.2.3 Synthesis of compound 3**

To maintain an argon atmosphere, place 60% sodium hydride (4.57 g, 114.36 mmol) in a double-mouth flask, add 50 ml of tetrahydrofuran solution, and stir continuously until the solution is homogeneous. At 0 °C, cyanomethyl phosphate (20.89 g, 18.48 ml, 114.36 mmol) was added dropwise. After the solution was clarified, compound 2 (15.00 g, 28.59 mmol) was dissolved in tetrahydrofuran solution and added to the system. Stir well and reflux overnight at 68 °C. The crude product obtained by the reaction was first extracted by ethyl acetate, the solvent was removed by a rotary evaporator, and the crude product was purified by column chromatography (in which the eluent petroleum ether:ethyl acetate (15:1 gradient to 10:1) was purified. Yellow oil 3 (10.37 g, 18.93 mmol) with a yield of 66.2 % was obtained. MS (MALDI) (M+, C_33_H_45_N_3_O_4_ ): calcd: 547.34; found : 547.39. ^1^H NMR (500 MHz, Chloroform-*d*) δ 7.37 (d, *J* = 8.8 Hz, 2H, ArH), 7.17 (d, *J* = 9.1 Hz, 2H, ArH), 6.72 (d, *J* = 8.4 Hz, 2H, ArH), 6.66 (d, *J* = 8.9 Hz, 2H, ArH), 5.35 (s, 1H, =CH), 4.63 – 4.58 (m, 2H, CH), 3.91 – 3.88 (m, 2H, OCH_2_), 3.84 – 3.80 (m, 2H, OCH_2_), 3.63 – 3.55 (m, 6H, CH_2_), 3.51 – 3.43 (m, 6H, CH_2_), 1.83 – 1.77 (m, 2H, OCH_2_), 1.74 – 1.68 (m, 2H, OCH_2_), 1.59 – 1.50 (m, 8H, NCH_2_), 1.19 (dt, *J* = 9.9, 7.0 Hz, 6H, CH_3_). ^13^C NMR (126 MHz, Chloroform-*d*) δ 163.09, 149.36, 149.06, 131.56, 131.54, 130.43, 130.40, 126.50, 124.22, 120.44, 110.96, 110.69, 99.16, 99.14, 86.22, 65.07, 64.96, 62.31, 62.28, 50.09, 50.05, 45.47, 45.43, 30.64, 30.61, 25.42, 25.39, 19.48, 19.45, 12.26, 12.18, 1.04.

**2.2.4 Synthesis of compound 4**

To maintain an argon atmosphere, slowly add diisobutylaluminum hydride solution (25.30 ml, 57.20 mmol) to compound 3 (10.37 g, 18.93 mmol) completely dissolved with dichloromethane at -78 °C, and after 3-4 hours of reaction, add 25 ml of the mixture of ethyl acetate and water, and continue the reaction at 0 °C for 2 hours, and the orange-yellow mixture was obtained after the reaction was completed. The crude product of the reaction was extracted with dichloromethane and water, and the lower organic layer was solvent removed by rotary evaporator. The crude product was purified by column chromatography, and a mixed ratio of petroleum ether to ethyl acetate (16:1 to 9:1) was used as an eluent. Compound 4 (4.53 g, 8.23 mmol) was obtained in the form of yellow oil with a yield of 43.48 %. MS (MALDI) (M+, C_33_H_46_N_2_O_5_): calcd: 550.34; found : 550.38. ^1^H NMR (500 MHz, Chloroform-*d*) δ 9.45 (d, *J* = 8.2 Hz, 1H, CHO), 7.28 (d, *J* = 9.0 Hz, 2H, ArH), 7.17 (d, *J* = 8.5 Hz, 2H, ArH), 6.70 (d, *J* = 8.3 Hz, 2H, ArH), 6.66 (d, *J* = 8.7 Hz, 2H, ArH), 6.39 (d, *J* = 8.2 Hz, 1H, =CH), 4.60 (dt, *J* = 14.5, 3.5 Hz, 2H, OCH), 3.94 – 3.87 (m, 2H, OCH_2_), 3.83 (tdd, *J* = 11.0, 8.1, 3.1 Hz, 2H, OCH_2_), 3.59 (qd, *J* = 11.9, 10.4, 8.4 Hz, 6H, CH_2_), 3.48 (tdd, *J* = 14.7, 6.3, 2.5 Hz, 6H, CH_2_), 1.80 (ddt, *J* = 15.4, 8.3, 4.1 Hz, 2H, CH_2_), 1.74 – 1.68 (m, 2H, CH_2_), 1.60 – 1.49 (m, 8H, CH_2_), 1.20 (dt, *J* = 14.1, 7.0 Hz, 6H, CH_3_). ^13^C NMR (126 MHz, Chloroform-*d*) δ 186.98, 170.58, 133.12, 131.13, 125.68, 111.66, 99.31, 65.03, 62.46, 30.74 (d, *J* = 3.5 Hz), 29.84, 25.51, 19.60 (d, *J* = 5.3 Hz), 12.27, 1.16.

**2.2.5 Synthesis of compound 5**

To maintain an argon atmosphere, place sodium ethanol (10 ml, 8.23 mmol) in a double-neck flask and add 2-mercaptoethanol (0.57 ml, 57.29 mmol). After stirring for 30 minutes at room temperature, isoforone oxide (1.27 g, 8.23 mmol) was added. After continuous stirring for 1 hour, add compound 4 (4.53 g, 8.23 mmol) dissolved in ultra-dry ethanol solvent and stir overnight at 65 °C. The reaction mixture was extracted with ethyl acetate, the organic layer was taken and the solvent was removed by rotary evaporation. The crude product was purified by column chromatography, and petroleum ether and ethyl acetate (15:1 to 2:1) were used as eluents to obtain red oily liquid compound 5 (2.66 g, 3.56 mmol) with a yield of 43.2 %. MS (MALDI) (M+, C_44_H_62_N_2_O_6_S): calcd: 746.43; found : 746.49. ^1^H NMR (500 MHz, Chloroform-*d*) δ 7.54 (d, *J* = 15.2 Hz, 1H, =CH_2_), 7.18 (d, *J* = 8.7 Hz, 2H, ArH), 7.08 (dd, *J* = 9.9, 3.3 Hz, 3H, ArH), 6.69 (dd, *J* = 10.1, 3.6 Hz, 3H, ArH), 6.58 (d, *J* = 8.6 Hz, 2H, =CH_2_), 4.56 (ddd, *J* = 17.7, 4.3, 2.9 Hz, 2H, OCH), 3.85 – 3.76 (m, 4H, NCH_2_), 3.59 – 3.41 (m, 14H, CH_2_), 2.76 (t, *J* = 5.6 Hz, 2H, CH_2_), 2.38 (s, 2H, CH_2_), 2.33 (s, 2 H, CH_2_), 1.76 (tt, *J* = 11.2, 5.7 Hz, 2H), 1.70 – 1.61 (m, 2H, CH_2_), 1.55 – 1.44 (m, 8H, CH_2_), 1.20 – 1.17 (m, 3H, CH_3_), 1.13 (t, *J* = 7.0 Hz, 3H, CH_3_), 0.95 (s, 6H, CH_3_). ^13^C NMR (126 MHz, Chloroform-*d*) δ 197.05, 160.47, 150.51, 148.07, 147.74, 138.88, 132.10, 129.85, 129.23, 129.20, 126.25, 126.18, 123.13, 110.93, 110.75, 98.96, 98.90, 64.92, 64.86, 62.12, 62.03, 60.13, 51.53, 49.94, 45.28, 45.22, 41.04, 38.54, 32.10, 30.48, 30.44, 28.11, 25.26, 25.24, 19.33, 19.27, 14.06, 12.13.

**2.2.6 Synthesis of compound 6**

To maintain an argon atmosphere, dissolve imidazole (0.32 g, 4.63 mmol), tert-butylchlorodiphenylsilane (1.27 g, 4.63 mmol), and compound 5 (2.66 g, 3.56 mmol) in 10 ml of dimethylformamide. After stirring at room temperature for 3 hours, the solvent is removed by rotational evaporation. The crude red oily product was purified by column chromatography, and the crude product was purified by the eluent petroleum ether:ethyl acetate (50:1 to 15:1) split. A yield of 99.5% red oily compound was obtained compound 6. MS (MALDI) (M+, C_62_H_81_N_3_O_5_SSi): calcd: 984.55; found : 984.59. ^1^H NMR (500 MHz, Chloroform-*d*) δ 7.58 – 7.55 (m, 4H, ArH), 7.30 – 7.27 (m, 4H, ArH), 7.12 (d, *J* = 5.1 Hz, 1H, =CH), 7.10 (d, *J* = 4.7 Hz, 2H, ArH), 7.04 (d, *J* = 8.4 Hz, 1H, =CH), 6.92 (dd, *J* = 15.3, 11.2 Hz, 1H, =CH), 6.67 – 6.59 (m, 4H, ArH), 6.58 – 6.53 (m, 4H, ArH), 4.52 (dt, *J* = 7.2, 3.8 Hz, 2H, CH), 3.83 – 3.73 (m, 6H, CH_2_), , 3.63 (t, *J* = 6.9 Hz, 3H, CH_2_), 3.44 – 3.36 (m, 10H, CH_2_), 2.84 (t, *J* = 6.9 Hz, 2H, CH_2_), 2.25 (s, 2H, CH_2_), 2.20 (s, 2H, CH_2_), 1.51 – 1.42 (m, 12H, CH_2_), 1.10 (t, *J* = 7.2 Hz, 6H, CH_3_), 0.96 (s, 9H, CH_3_), 0.86 (s, 6H, CH_3_). ^13^C NMR (126 MHz, Chloroform-*d*) δ 191.72, 150.63, 135.56, 134.82, 133.58, 132.16, 131.88, 129.72, 129.69, 128.36, 127.75, 126.76, 124.65, 111.81, 63.17, 60.51, 60.22, 60.17, 52.51, 45.66, 45.64, 41.48, 39.86, 37.23, 29.98, 28.24, 26.91, 21.12, 19.26, 14.25, 12.06, 12.04.

**2.2.7 Synthesis of compound 7**

The synthesis steps are the same as for compound 3. The crude product was purified by column chromatography with the eluents petroleum ether and ethyl acetate (15:1 to 10:1). A red oil with a yield of 67.4 % was obtained compound 7. MS (MALDI) (M+, C_62_H_81_N_3_O_5_SSi): calcd: 1007.57; found : 1007.59. ^1^H NMR (500 MHz, Chloroform-*d*) δ 7.65 (dd, 4H, ArH), 7.48 (d, *J* = 15.2 Hz, 1H, =CH), 7.41 – 7.35 (m, 6H, ArH), 7.19 (d, *J* = 8.8 Hz, 2H, ArH), 7.11 (d, *J* = 8.3 Hz, 2H, ArH), 6.86 (dd, *J* = 15.3, 11.3 Hz, 1H, ArH), 6.76 – 6.72 (m, 2H, =CH), 6.67 – 6.61 (m, 3H, ArH), 6.17 (s, 1H, =CH), 4.66 – 4.60 (m, 2H, OCH), 3.91 – 3.81 (m, 4H, OCH_2_), 3.70 (t, *J* = 6.8 Hz, 2H, OCH_2_), 3.66 – 3.56 (m, 6H, CH_2_), 3.53 – 3.46 (m, 6H, CH_2_), 2.70 (t, *J* = 6.8 Hz, 2H, CH_2_), 2.44 (s, 2H, CH_2_), 2.20 (s, 2H, CH_2_), 1.86 – 1.65 (m, 6H, CH_2_), 1.59 – 1.52 (m, 6 H, CH_2_), 1.24 (t, *J* = 7.0 Hz, 3H, CH_3_), 1.19 (t, *J* = 7.0 Hz, 3H, CH_3_), 1.06 (d, *J* = 5.5 Hz, 9H, CH_3_), 0.95 (d, *J* = 16.2 Hz, 2H, CH_3_), 0.89 (s, 3H, CH_3_), 0.86 (s, 1H, CH_3_). ^13^C NMR (126 MHz, Chloroform-*d*) δ 158.55, 149.58, 147.93, 147.83, 147.69, 135.63, 133.55, 132.21, 129.86, 127.85, 111.18, 111.04, 99.30, 99.25, 94.43, 65.23, 65.18, 63.05, 62.47, 62.39, 50.22, 45.54, 45.48, 43.49, 41.51, 37.57, 30.76, 30.73, 30.15, 28.01, 26.96, 25.51, 19.63, 19.58, 12.39.

**2.2.8 Synthesis of compound 8**

Following the steps of synthetic compound 4, a red oil with a yield of 42.16% was obtained compound 8. MS (MALDI) (M+, C_62_H_82_N_2_O_6_SSi): calcd: 1010.57; found : 1010.59. ^1^H NMR (500 MHz, Chloroform-*d*) δ 10.08 (d, *J* = 8.1 Hz, 1H, CHO), 7.66 – 7.63 (m, 4H, ArH), 7.59 (d, *J* = 15.2 Hz, 1H, =CH), 7.38 (d, *J* = 6.9 Hz, 2H, ArH), 7.36 (d, 2H, ArH), 7.37 (d, 2H, ArH), 7.22 (d, *J* = 8.6 Hz, 2H, ArH), 7.11 (d, *J* = 8.5 Hz, 2H, ArH), 6.95 (d, *J* = 8.1 Hz, 1H, =CH), 6.88 (dd, *J* = 15.2, 11.3 Hz, 1H, =CH), 6.75 (d, *J* = 8.4 Hz, 2H, ArH), 6.67 (d, *J* = 11.2 Hz, 1H, =CH), 6.63 (d, *J* = 8.7 Hz, 2H, ArH), 4.63 (ddd, *J* = 17.2, 4.4, 3.0 Hz, 2H, OCH), 3.99 – 3.94 (m, 1H, OCH_2_), 3.91 – 3.89 (m, 1H, OCH_2_), 3.88 – 3.82 (m, 2H, OCH_2_), 3.72 (t, *J* = 7.0 Hz, 2H, OCH_2_), 3.66 – 3.57 (m, 6H, CH_2_), 3.53 – 3.45 (m, 6H, CH_2_), 2.72 (t, *J* = 7.0 Hz, 2H, SCH_2_), 2.59 (s, 2H, CH_2_), 2.23 (s, 2H, CH_2_), 1.86 – 1.81 (m, 2H, CH_2_), 1.73 (dt, *J* = 12.3, 3.3 Hz, 2H, CH_2_), 1.61 – 1.52 (m, 8H, CH_2_), 1.25 (t, *J* = 7.0 Hz, 3H, CH_3_), 1.20 (t, *J* = 7.0 Hz, 3H, CH_3_), 1.06 (s, 9H, CH_3_), 0.91 (s, 6H, CH_3_). ^13^C NMR (126 MHz, Chloroform-*d*) δ 191.59, 156.66, 150.66, 147.91, 135.62, 133.66, 132.22, 129.75, 127.79, 126.95, 126.76, 124.29, 111.18, 111.04, 99.28, 99.23, 65.20 (d, *J* = 7.3 Hz), 63.26, 62.40 (d, *J* = 9.9 Hz), 50.22, 45.50 (d, *J* = 7.4 Hz), 41.53, 39.94, 37.27, 30.73 (d, *J* = 3.8 Hz), 30.02, 28.28, 26.95, 25.50 (d, *J* = 1.8 Hz), 19.59 (d, *J* = 6.1 Hz), 19.31, 12.39 (d, *J* = 1.8 Hz).

**2.2.8 Synthesis of compound 9**

Dissolve compound 8 (1.02 g, 1.01 mmol) in 6 ml of acetone, add 8 ml of concentrated hydrochloric acid (1 M) to this solution, react for 3 hours at room temperature, and then use dichloromethane for extraction. The crude product was purified by column chromatography, and petroleum ether and ethyl acetate (15:1 to 1:1) were used as eluents to obtain red solid compound 9 (0.36 g, 0.43 mmol) with a yield of 42.6 %. MS (MALDI) (M+, C_52_H_66_N_2_O_4_SSi): calcd: 842.45; found : 842.49. ^1^H NMR (500 MHz, Chloroform-*d*) δ 10.06 (d, *J* = 8.1 Hz, 1H, CHO), 7.65 (d, *J* = 7.5 Hz, 4H, ArH), 7.60 (s, 1H, =CH), 7.37 (dd, *J* = 11.3, 6.8 Hz, 6H, ArH), 7.20 (d, *J* = 8.4 Hz, 2H, ArH), 7.16 (d, *J* = 8.2 Hz, 2H, ArH), 6.92 (d, *J* = 8.1 Hz, 1H, =CH), 6.90 – 6.83 (m, 1H, =CH), 6.80 (d, *J* = 8.3 Hz, 2H, ArH), 6.70 (s, 1H, =CH), 6.69 – 6.66 (m, 2H), 3.84 (dt, *J* = 26.9, 6.2 Hz, 4H, NCH_2_), 3.73 (t, *J* = 7.0 Hz, 2H, OCH_2_), 3.56 (t, *J* = 6.1 Hz, 2H, CH_2_), 3.51 (d, *J* = 6.4 Hz, 4H, NCH_2_), 3.45 (t, *J* = 7.2 Hz, 2H, CH_2_), 2.73 (t, *J* = 7.0 Hz, 2H, SCH_2_), 2.58 (s, 2H, CH_2_), 2.22 (s, 2H, CH_2_), 2.16 (s, 3H, OH), 2.05 (s, 1H, OH), 1.23 (t, *J* = 7.2 Hz, 3H, CH_3_), 1.19 (d, *J* = 7.1 Hz, 3H, CH_3_), 1.06 (s, 9H, CH_3_), 0.90 (s, 6H, CH_3_). ^13^C NMR (126 MHz, Chloroform-*d*) δ 191.72, 156.85, 150.63, 148.12, 147.92, 147.34, 135.56, 134.82, 133.58, 132.16, 131.88, 129.72, 129.69, 128.36, 127.75, 126.76, 124.65, 111.81, 63.17, 60.51, 60.22, 60.17, 52.51, 45.65, 41.48, 39.86, 37.23, 29.98, 28.24, 26.91, 21.12, 19.26, 14.25, 12.06, 12.04

**2.2.9 Synthesis of the 10a-f series of compounds**

**Compound 10a：**Functional groups R1 (0.53 g, 1.72 mmol), DMAP (0.02 g, 0.17 mmol) and EDCI (0.32 g, 1.72 mmol) were dissolved in 15 ml of ultra-dry dichloromethane in an argon-gas and 0 °C atmosphere, and the solution gradually became turbid. After stirring for about 45 min, it became clear, and 9 compounds dissolved by dichloromethane (0.36 g, 0.43 mmol) were added. After holding the reaction at 0 °C for 2 h, the solution was raised to 40 °C for condensation and reflux reaction for 15 h. The solution is extracted using methylene chloride, the solvent is removed by a rotary evaporator, and the crude product is purified by column chromatography (in which the eluent petroleum ether:ethyl acetate (20:1 gradient to 2:1)) is purified. A red solid compound 10a with a yield of 52.6 % was obtained. MS (MALDI) (M+, C_86_H_90_N_2_O_6_SSi): calcd: 1306.63; found : 1306.65. ^1^H NMR (500 MHz, Chloroform-*d*) δ 10.07 (d, *J* = 8.0 Hz, 1H, CHO), 8.36 (d, *J* = 10.3 Hz, 2H, ArH), 8.23 (d, *J* = 8.6 Hz, 4H, ArH), 8.00 (t, *J* = 8.3 Hz, 4H, ArH), 7.67 (d, *J* = 6.8 Hz, 4H, ArH), 7.54 (d, *J* = 15.2 Hz, 1H, =CH), 7.50 (d, *J* = 8.7 Hz, 4H, ArH), 7.46 (d, *J* = 7.5 Hz, 4H, ArH), 7.37 (dd, *J* = 12.1, 7.1 Hz, 6H, ArH), 7.16 (d, *J* = 8.5 Hz, 2H, ArH), 7.09 (d, *J* = 8.2 Hz, 2H, ArH), 6.93 (d, *J* = 8.0 Hz, 1H, =CH), 6.78 (t, *J* = 13.2 Hz, 1H, =CH), 6.69 (d, *J* = 8.3 Hz, 2H, ArH), 6.64 (d, *J* = 11.3 Hz, 1H, =CH), 6.57 (d, *J* = 8.4 Hz, 2H, ArH), 4.29 (dt, *J* = 30.1, 6.4 Hz, 4H, OCH_2_), 3.95 (t, *J* = 8.4 Hz, 4H, NCH_2_), 3.73 (t, *J* = 7.1 Hz, 2H, OCH_2_), 3.51 (dt, *J* = 32.4, 6.4 Hz, 4H, NCH_2_), 3.38 (dt, *J* = 24.7, 7.2 Hz, 4H, CH_2_), 2.79 (t, *J* = 8.4 Hz, 4H, CH_2_), 2.72 (t, *J* = 7.1 Hz, 2H, CH_2_), 2.50 (s, 2H, CH_2_), 2.09 (s, 2H, CH_2_), 1.18 (dt, *J* = 25.4, 7.1 Hz, 6H, CH_3_), 1.06 (s, 9H, CH_3_), 0.83 (s, 6H, CH_3_). ^13^C NMR (126 MHz, Chloroform-*d*) δ 191.66, 173.12, 156.60, 150.53, 147.59, 147.35, 135.65, 134.75, 133.68, 132.31, 132.28, 132.20, 131.91, 131.68, 131.66, 130.55, 129.78, 129.59, 129.46, 128.41, 127.82, 127.47, 126.85, 126.57, 126.15, 126.12, 125.09, 124.71, 123.95, 123.88, 111.35, 111.19, 63.28, 60.53, 48.66, 41.43, 39.86, 37.31, 35.41, 35.39, 29.94, 28.22, 26.97, 23.37, 23.35, 19.33, 14.32, 12.35.

**Compound 10b：**The synthesis step was the same as that of 11a, and the functional group R2 (0.55 g, 1.72 mmol) and compound 9 (0.36 g, 0.43 mmol) were used to prepare red solid compound 10b with a yield of 84.5 % (0.48 g, 0.36 mmol). MS (MALDI) (M+, C_78_H_82_N_2_O_14_SSi): calcd: 1330.53; found : 1330.56. ^1^H NMR (500 MHz, Chloroform-*d*) δ 10.07 (d, *J* = 8.2 Hz, 1H, CHO), 7.73 (d, *J* = 11.7 Hz, 4H, ArH), 7.63 (d, *J* = 9.8 Hz, 4H, ArH), 7.61 (d, *J* = 20.7 Hz, 1H, =CH), 7.38 (d, *J* = 7.6 Hz, 2H, ArH), 7.35 (t, *J* = 7.2 Hz, 4H, ArH), 7.26 – 7.23 (m, 2H, ArH), 7.19 (d, *J* = 8.4 Hz, 2H, ArH), 7.13 (d, *J* = 8.3 Hz, 2H, ArH), 6.95 (d, *J* = 8.1 Hz, 1H, =CH), 6.86 (dd, *J* = 15.1, 11.3 Hz, 1H, =CH), 6.80 (d, *J* = 8.3 Hz, 2H, ArH), 6.70 – 6.67 (m, 2H, ArH), 6.63 (d, *J* = 6.9 Hz, 2H, ArH), 6.60 (d, *J* = 7.2 Hz, 2H, ArH), 6.31 (d, *J* = 9.0 Hz, 2H, ArH), 6.27 (d, *J* = 9.5 Hz, 2H, ArH), 6.06 (s, 1H, =CH), 6.03 (dd, *J* = 10.2, 4.7 Hz, 4H, ArH), 4.51 (dt, *J* = 22.8, 6.5 Hz, 4H, NCH_2_), 3.74 (dt, *J* = 24.9, 6.8 Hz, 6H, OCH_2_), 3.50 (dq, *J* = 26.1, 7.1 Hz, 4H, NCH_2_), 2.72 (t, *J* = 7.0 Hz, 2H, SCH_2_), 2.58 (s, 2H, CH_2_), 2.22 (s, 2H, CH_2_), 1.21 (t, *J* = 7.0 Hz, 6H, CH_3_), 1.05 (s, 9H, CH_3_), 0.89 (s, 6H, CH_3_). ^13^C NMR (126 MHz, Chloroform-*d*) δ 191.78, 165.03, 164.97, 164.02, 163.98, 151.08, 151.05, 135.66, 134.88, 133.71, 133.68, 133.57, 132.37, 132.17, 131.95, 130.71, 129.88, 129.80, 128.50, 127.83, 127.63, 127.47, 127.44, 127.38, 126.89, 124.74, 120.52, 120.51, 111.51, 111.36, 63.28, 48.77, 45.55, 37.31, 28.31, 26.98, 19.35, 12.55, 12.52, 1.15.

**Compound 10c：**The synthesis step was the same as that of 10a, and the red solid compound 10c was prepared by using functional group R3 (0.34 g, 1.72 mmol) and compound 9 (0.36 g, 0.43 mmol) with a yield of 42.2 % (0.20 g, 0.18 mmol). MS (MALDI) (M+, C_66_H_78_N_2_O_8_SSi): calcd: 1086.5; found : 1086.54. ^1^H NMR (500 MHz, Chloroform-*d*) δ 10.07 (d, *J* = 8.0 Hz, 1H, CHO), 7.65 – 7.63 (m, 4H, ArH), 7.60 (d, *J* = 15.3 Hz, 1H, =CH), 7.40 – 7.35 (m, 4H, ArH), 7.35 – 7.29 (m, 4H, ArH), 7.20 (d, *J* = 8.3 Hz, 2H, ArH), 7.15 (d, *J* = 8.1 Hz, 2H, ArH), 6.95 (d, *J* = 8.1 Hz, 1H, =CH)), 6.85 (dd, *J* = 15.3, 11.2 Hz, 1H, =CH)), 6.74 (d, *J* = 8.3 Hz, 2H, ArH), 6.67 (d, *J* = 11.3 Hz, 1H, =CH)), 6.63 (d, *J* = 8.4 Hz, 2H, ArH), 6.29 – 6.26 (m, 2H, ArH), 6.05 – 6.01 (m, 2H, ArH), 4.29 (dt, *J* = 26.1, 6.3 Hz, 4H, OCH_2_), 3.72 (t, *J* = 6.9 Hz, 2H, OCH_2_), 3.59 (dt, *J* = 26.6, 6.4 Hz, 4H, NCH_2_), 3.44 (dq, *J* = 26.7, 7.0 Hz, 4H, NCH_2_), 2.99 – 2.95 (m, 4H, CH_2_), 2.72 – 2.70 (m, 2H, CH_2_), 2.69 – 2.63 (m, 4H, CH_2_), 2.58 (s, 2H, CH_2_), 2.21 (s, 2H, CH_2_), 1.21 (dt, *J* = 25.9, 7.0 Hz, 6H, CH_3_), 1.05 (s, 9H, CH_3_), 0.90 (s, 6H, CH_3_). ^13^C NMR (126 MHz, Chloroform-*d*) δ 191.75, 177.04, 172.63, 172.59, 156.76, 154.05, 154.02, 153.94, 150.59, 147.59, 147.31, 141.42, 141.40, 141.38, 135.65, 134.79, 133.67, 132.31, 132.00, 130.58, 129.79, 128.50, 127.81, 127.50, 126.89, 124.73, 111.40, 111.25, 110.34, 105.54, 105.51, 63.26, 61.95, 61.86, 48.80, 45.44, 41.55, 39.95, 37.30, 32.80, 32.76, 32.34, 30.05, 28.30, 26.97, 23.52, 23.49, 19.33, 12.42.

**Compound 10d：**The synthesis step was the same as that of 10a, and the red solid compound was prepared from compound R4 (0.39 g, 1.72 mmol) and compound 9 (0.36 g, 0.43 mmol) for copound 10d with a yield of 63.7 % (0.31 g, 0.27 mmol). MS (MALDI) (M+, C_66_H_76_N_4_O_10_SSi): calcd: 1144.51; found : 1144.55. ^1^H NMR (500 MHz, Chloroform-*d*) δ 10.06 (d, *J* = 5.7 Hz, 1H, CHO), 7.63 (d, *J* = 7.1 Hz, 4H, ArH), 7.58 (s, 1H, =CH), 7.36 (dd, *J* = 11.9, 6.8 Hz, 6H, ArH), 7.35 (s, 1H, =CH), 7.17 (d, *J* = 8.2 Hz, 2H, ArH), 7.13 (d, *J* = 8.3 Hz, 2H, ArH), 6.92 (d, *J* = 8.1 Hz, 1H, =CH), 6.87 – 6.80 (m, 1H, =CH), 6.73 (d, *J* = 8.5 Hz, 2H, ArH), 6.69 (dd, *J* = 9.4, 2.4 Hz, 4H, ArH), 6.62 (d, *J* = 8.6 Hz, 2H, ArH), 4.26 (dt, *J* = 25.4, 6.9 Hz, 4H, NCH_2_), 3.86 – 3.79 (m, 4H, NCH_2_), 3.71 (t, *J* = 6.7 Hz, 2H, OCH_2_), 3.59 (dt, *J* = 25.9, 6.9 Hz, 4H, OCH_2_), 3.45 (dq, *J* = 26.6, 7.3 Hz, 4H, NCH_2_), 2.71 (t, *J* = 7.4 Hz, 2H, SCH_2_), 2.68 – 2.62 (m, 4H, CH_2_), 2.57 (s, 2H, CH_2_), 2.21 (s, 2H, CH_2_), 1.23 (t, 3H, CH_3_), 1.18 (t, *J* = 6.6 Hz, 3H, CH_3_), 1.04 (s, 9H, CH_3_), 0.90 (s, 6H, CH_3_). ^13^C NMR (126 MHz, Chloroform-*d*) δ 191.71, 170.79, 170.77, 170.42, 156.70, 150.55, 147.58, 147.28, 135.65, 134.36, 134.32, 133.68, 132.31, 132.04, 129.80, 129.78, 127.82, 126.91, 124.77, 111.42, 111.29, 63.27, 62.10, 48.71, 45.42, 41.56, 39.95, 37.31, 33.69, 33.52, 33.03, 30.06, 28.31, 26.97, 19.34, 12.45, 12.41.

**Compound 10e：**In the same synthesis step as 10a, the ratio of the amount of compound 9 (0.36 g, 0.43 mmol) to R5 (0.38 g, 1.72 mmol) was 1:4, and the red solid compound 10e (0.33 g, 0.29 mmol) was prepared with a yield of 67.9%. MS (MALDI) (M+, C_66_H_72_N_8_O_6_SSi): calcd: 1132.51; found : 1132.59. ^1^H NMR (500 MHz, Chloroform-*d*) δ 10.05 (d, *J* = 8.1 Hz, 1H, CHO), 8.03 (dd, *J* = 17.0, 8.4 Hz, 4H, ArH), 7.66 – 7.63 (m, 4H, ArH), 7.38 (d, *J* = 6.5 Hz, 2H, ArH), 7.36 (d, *J* = 7.1 Hz, 4H, ArH), 7.33 (d, *J* = 1.8 Hz, 1H, =CH), 7.21 (d, *J* = 8.5 Hz, 2H, ArH), 7.16 (d, *J* = 8.3 Hz, 2H, ArH), 7.06 (dd, *J* = 8.4, 5.1 Hz, 4H, ArH), 6.93 (d, *J* = 8.1 Hz, 1H, =CH), 6.90 – 6.85 (m, 1H, =CH), 6.82 (t, *J* = 6.9 Hz, 2H, ArH), 6.70 (d, *J* = 8.3 Hz, 2H, ArH), 6.67 (s, 1H, =CH), 4.52 (dt, *J* = 24.6, 6.3 Hz, 4H, OCH_2_), 3.79 (t, *J* = 6.3 Hz, 2H, OCH_2_), 3.76 – 3.71 (m, 4H, NCH_2_), 3.55 (q, *J* = 7.1 Hz, 2H, NCH_2_), 3.50 (q, *J* = 7.1 Hz, 2H, NCH_2_), 2.72 (t, *J* = 7.0 Hz, 2H, SCH_2_), 2.58 (s, 2H, CH_2_), 2.22 (s, 2H, CH_2_), 1.28 (t, *J* = 7.0 Hz, 3H, CH_3_), 1.23 (t, *J* = 7.0 Hz, 3H, CH_3_), 1.05 (s, 9H, CH_3_), 0.89 (s, 6H, CH_3_). ^13^C NMR (126 MHz, Chloroform-*d*) δ 191.65, 165.92, 165.86, 156.58, 150.48, 147.68, 147.38, 147.22, 145.09, 145.03, 135.64, 134.70, 133.67, 132.32, 132.13, 131.46, 130.57, 129.76, 129.65, 128.46, 127.79, 127.48, 126.86, 126.46, 124.68, 119.11, 118.99, 111.54, 111.35, 63.26, 62.57, 62.42, 48.89, 45.47, 41.55, 39.93, 37.30, 30.03, 28.30, 27.02, 26.96, 19.33, 12.53, 12.51.

**Compound 10f：**In the same synthesis step as 10a, the ratio of the amount of compound 9 (0.36 g, 0.43 mmol) to R6 (0.50 g, 1.72 mmol) was 1:4, and the red solid compound 10f (0.30 g, 0.24 mmol) was prepared with a yield of 55.8 %. MS (MALDI) (M+, C_78_H_82_N_2_O_10_SSi): calcd: 1266.55; found : 1266.59. ^1^H NMR (500 MHz, Chloroform-*d*) δ 10.01 (d, *J* = 8.1 Hz, 1H, CHO), 7.57 (d, *J* = 7.3 Hz, 4H, ArH), 7.54 (s, 1H, =CH), 7.34 (d, *J* = 7.2 Hz, 1H, =CH), 7.30 (q, *J* = 7.3, 6.1 Hz, 6H, ArH), 7.24 – 7.19 (m, 4H, ArH), 7.16 (d, *J* = 8.4 Hz, 2H, ArH), 7.11 (d, *J* = 8.3 Hz, 2H, ArH), 6.87 (d, *J* = 8.2 Hz, 1H, =CH), 6.81 (d, *J* = 11.0 Hz, 1H, =CH), 6.77 (s, 2H, ArH), 6.74 (d, 2H, ArH), 6.63 (d, *J* = 11.1 Hz, 2H, ArH), 4.65 (d, *J* = 7.5 Hz, 8H, OCH_2_), 4.47 (dt, *J* = 23.9, 6.5 Hz, 4H, NCH_2_), 3.70 (dt, *J* = 29.0, 6.9 Hz, 6H, OCH_2_), 3.54 – 3.44 (m, 4H, NCH_2_), 2.67 (t, *J* = 7.0 Hz, 2H, SCH_2_), 2.49 (s, 4H, ≡CH), 1.56 (s, 4H, CH_2_), 1.23 (t, 3H, CH_3_), 1.17 (t, 3H, CH_3_), 1.00 (s, 9H, CH_3_), 0.84 (s, 6H, CH_3_). ^13^C NMR (126 MHz, Chloroform-*d*) δ 191.68, 166.12, 158.69, 158.66, 156.60, 135.67, 133.70, 132.38, 132.04, 129.86, 129.80, 127.84, 126.97, 109.14, 109.07, 107.84, 107.74, 78.07, 78.04, 76.20, 76.18, 63.29, 56.27, 41.57, 39.96, 37.33, 30.07, 29.84, 28.33, 26.99, 19.36, 12.52.

**2.2.10 Synthesis of Chromophore YZ1-YZ6**

**Chromophore YZ1：**Maintaining an argon atmosphere, compound 10a (0.30 g, 0.23 mmol) as well as acceptor CF_3_-TCF (0.08 g, 0.27 mmol) were dissolved in 5 ml of absolute ethanol and refluxed at 65 °C for 3 h. The blue-purple solid chromophore YZ1 (0.19 g, 0.12 mmol) was obtained by column chromatography with petroleum ether and ethyl acetate (ratios ranging from 12:1 to 3:1) as eluents under vacuum conditions with a yield of 52.1 %. HRMS (ESI) (M+, C_102_H_96_F_3_N_5_O_6_SSi ): calcd: 1603.6803; found: 1603.68035. ^1^H NMR (500 MHz, Chloroform-*d*) δ 8.35 (d, *J* = 15.1 Hz, 2H, ArH), 8.20 (t, *J* = 8.5 Hz, 4H, ArH), 8.08 (d, *J* = 25.6 Hz, 1H, =CH), 8.02 – 7.96 (m, 4H, ArH), 7.61 (d, *J* = 7.5 Hz, 4H, ArH), 7.56 (s, 1H, =CH), 7.50 (t, *J* = 7.7 Hz, 4H, ArH), 7.47 (d, *J* = 4.1 Hz, 5H, ArH), 7.46 – 7.41 (m, 4H, ArH), 7.39 (d, *J* = 6.5 Hz, 1H, =CH), 7.36 (d, *J* = 7.4 Hz, 2H, ArH), 7.32 (d, *J* = 7.3 Hz, 3H, ArH), 7.30 (s, 1H, =CH), 7.16 (d, *J* = 8.4 Hz, 2H, ArH), 7.07 (d, *J* = 8.2 Hz, 2H, ArH), 6.96 – 6.90 (m, 1H, =CH), 6.67 (d, *J* = 9.5 Hz, 3H, ArH), 6.56 (d, *J* = 8.5 Hz, 2H, ArH), 6.37 (d, *J* = 14.4 Hz, 1H, =CH), 4.28 (dt, *J* = 30.2, 6.4 Hz, 4H, OCH_2_), 3.93 (t, *J* = 8.3 Hz, 4H, NCH_2_), 3.69 (t, *J* = 6.8 Hz, 2H, OCH_2_), 3.51 (dt, *J* = 30.9, 6.5 Hz, 4H, NCH_2_), 3.43 – 3.33 (m, 4H, CH_2_), 2.80 – 2.73 (m, 4H, CH_2_), 2.69 (t, *J* = 6.9 Hz, 2H, SCH_2_), 2.05 (s, 4H, CH_2_), 1.19 (t, *J* = 7.0 Hz, 3H, CH_3_), 1.13 (t, *J* = 7.2 Hz, 3H, CH_3_), 1.04 (s, 9H, CH_3_), 0.76 (s, 3H, CH_3_), 0.70 (s, 3H, CH_3_). ^13^C NMR (126 MHz, Chloroform-*d*) δ 175.96, 173.09, 162.12, 147.18, 135.60, 133.52, 132.71, 132.26, 132.15, 131.68, 131.66, 131.31, 130.58, 129.88, 129.70, 129.58, 129.57, 129.48, 128.46, 127.84, 126.82, 126.59, 126.18, 126.13, 125.10, 123.93, 123.84, 116.71, 111.70, 111.39, 111.20, 62.92, 41.53, 41.15, 38.32, 35.40, 35.36, 30.26, 29.83, 28.35, 27.83, 26.93, 23.36, 23.32, 19.35, 12.33.

**Chromophore YZ2：**Compound 10b (0.48 g, 0.36 mmol) was reacted with the CF_3_-TCF acceptor(0.19 g, 0.61 mmol) (steps as above) to prepare blue-purple solid chromophore YZ2 with a yield of 48.6 % (0.28 g, 0.17 mmol). HRMS (ESI) (M+, C_94_H_88_F_3_N_5_O_14_SSi ): calcd: 1627.5770; found: 1627.5772. ^1^H NMR (500 MHz, Chloroform-*d*) δ 8.08 (d, *J* = 28.2 Hz, 1H, =CH), 7.72 (d, *J* = 12.8 Hz, 4H), 7.65 (d, *J* = 15.2 Hz, 1H), 7.62 (d, *J* = 7.3 Hz, 4H), 7.51 (d, *J* = 8.6 Hz, 1H), 7.49 (d, *J* = 4.4 Hz, 4H), 7.41 (d, *J* = 12.5 Hz, 1H), 7.37 (d, *J* = 7.5 Hz, 2H), 7.32 (t, *J* = 7.4 Hz, 4H), 7.25 (s, 2H), 7.22 (d, *J* = 8.6 Hz, 2H), 7.15 (d, *J* = 8.3 Hz, 2H), 7.09 – 7.02 (m, 1H), 6.80 (d, *J* = 8.3 Hz, 2H), 6.77 (d, *J* = 11.3 Hz, 1H), 6.67 (d, *J* = 8.4 Hz, 2H), 6.62 (dd, *J* = 17.3, 4.0 Hz, 4H), 6.35 (d, *J* = 14.3 Hz, 1H), 6.32 (ddd, *J* = 16.8, 10.3, 5.3 Hz, 4H), 6.05 (d, *J* = 10.4 Hz, 4H), 4.51 (dt, *J* = 20.8, 6.5 Hz, 4H, OCH_2_), 3.78 (t, 2H, OCH_2_), 3.71 (dt, *J* = 13.2, 6.6 Hz, 4H, CH_2_), 3.51 (dq, *J* = 22.3, 7.1 Hz, 4H, NCH_2_), 2.75 (t, *J* = 6.4 Hz, 2H, SCH_2_), 1.64 (s, 4H, CH_2_), 1.24 (t, *J* = 7.3 Hz, 9H, CH_3_), 0.85 (s, 3H, CH_3_), 0.78 (s, 3H, CH_3_). ^13^C NMR (126 MHz, Chloroform-*d*) δ 175.95, 165.02, 164.96, 163.97, 162.24, 151.11, 151.07, 147.24, 135.62, 133.74, 133.70, 133.52, 132.81, 132.09, 131.33, 130.65, 130.31, 129.90, 129.72, 128.79, 127.85, 127.43, 127.38, 126.84, 120.52, 120.43, 111.52, 111.30, 111.16, 62.93, 48.76, 45.64, 41.71, 41.27, 38.33, 32.06, 30.40, 29.83, 26.94, 19.36, 14.27, 12.54.

**Chromophore YZ3：**Compound 10c (0.20 g, 0.18 mmol) was reacted with CF₃-TCF acceptor(0.10 g, 0.31 mmol) (steps are the same as above) to prepare blue-violet solid chromophore YZ3 with a yield of 49.4 % (0.12 g, 0.09 mmol). HRMS (ESI) (M+, C_82_H_84_F_3_N_5_O_8_SSi ): calcd: 1383.5762; found: 1383.5766. ^1^H NMR (500 MHz, Chloroform-*d*) δ 8.03 (s, 1H, =CH), 7.64 (d, *J* = 15.3 Hz, 2H, ArH), 7.60 (d, *J* = 7.3 Hz, 4H, ArH), 7.50 (s, 1H, =CH), 7.48 (s, 5H, ArH), 7.41 (d, *J* = 12.7 Hz, 1H, =CH), 7.33 (dd, *J* = 19.7, 7.5 Hz, 6H, ArH), 7.22 (d, *J* = 2.7 Hz, 1H, =CH), 7.19 (d, *J* = 8.3 Hz, 2H, ArH), 7.14 (d, *J* = 8.3 Hz, 2H, ArH), 7.06 (d, *J* = 12.7 Hz, 1H, =CH), 6.75 (d, *J* = 11.1 Hz, 2H, =CH), 6.65 (d, *J* = 8.4 Hz, 2H, =CH), 6.38 (d, *J* = 14.3 Hz, 1H, =CH), 6.26 (s, 2H, ArH), 5.96 (d, *J* = 5.2 Hz, 2H, ArH), 4.24 (dt, *J* = 24.0, 6.5 Hz, 4H, OCH_2_), 3.67 (d, *J* = 7.0 Hz, 2H, OCH_2_), 3.58 (dt, *J* = 22.8, 6.7 Hz, 4H, NCH_2_), 3.44 (dt, *J* = 23.8, 7.3 Hz, 4H, NCH_2_), 2.93 (dd, *J* = 12.1, 7.1 Hz, 4H, CH_2_), 2.66 (dt, *J* = 22.9, 7.4 Hz, 6H, CH_2_), 2.24 (s, 2H, CH_2_), 2.02 (s, 1H, CH_2_), 1.66 (s, 1H, CH_2_), 1.22 (t, *J* = 11.4 Hz, 3H, CH_3_), 1.16 (t, 3H, CH_3_), 1.01 (s, 9H, CH_3_), 0.85 (s, 3H, CH_3_), 0.74 (s, 3H, CH_3_). ^13^C NMR (126 MHz, Chloroform-*d*) δ 175.92, 172.55, 172.51, 162.08, 158.57, 155.16, 153.98, 153.96, 151.58, 148.33, 147.92, 147.18, 141.36, 135.56, 133.48, 132.73, 132.11, 131.28, 130.67, 130.58, 130.28, 129.99, 129.84, 129.67, 128.76, 127.79, 126.99, 126.79, 125.18, 116.68, 112.04, 111.68, 111.40, 111.18, 110.32, 105.52, 105.49, 62.88, 61.76, 61.69, 48.74, 45.46, 41.65, 41.22, 38.29, 32.75, 32.71, 32.00, 31.59, 30.34, 29.77, 28.40, 27.87, 26.89, 23.48, 23.45, 19.31, 12.39.

**Chromophore YZ4：**The blue-purple solid chromophore YZ4 was prepared by reacting compound 10d (0.31 g, 0.27 mmol) with CF_3_-TCF acceptor(0.15 g, 0.46 mmol) (steps as above). HRMS (ESI) (M+, C_82_H_82_F_3_N_7_O_10_SSi ): calcd: 1441.5565; found: 1441.5566. ^1^H NMR (500 MHz, Chloroform-*d*) δ 8.06 (s, 1H), 7.65 (d, *J* = 15.7 Hz, 1H), 7.61 (d, *J* = 7.3 Hz, 4H), 7.51 (d, *J* = 8.5 Hz, 1H), 7.49 – 7.46 (m, 4H), 7.42 (d, *J* = 12.6 Hz, 1H), 7.37 (d, *J* = 7.3 Hz, 2H), 7.30 (t, *J* = 7.4 Hz, 4H), 7.25 (d, *J* = 8.3 Hz, 2H), 7.16 (d, *J* = 8.4 Hz, 2H), 7.05 (t, *J* = 13.1 Hz, 1H), 6.72 (d, *J* = 10.1 Hz, 3H), 6.72 – 6.66 (m, 4H), 6.61 (d, *J* = 8.4 Hz, 2H), 6.35 (d, *J* = 14.3 Hz, 1H), 4.24 (dt, *J* = 23.1, 6.4 Hz, 4H, NCH_2_), 3.85 (dt, *J* = 13.5, 7.1 Hz, 4H, OCH_2_), 3.70 (t, *J* = 7.0 Hz, 2H, OCH_2_), 3.61 (dt, *J* = 22.0, 6.7 Hz, 4H, NCH_2_), 3.45 (dt, *J* = 22.9, 7.2 Hz, 4H, CH_2_), 2.70 (t, *J* = 8.2 Hz, 2H, SCH_2_), 2.68 – 2.62 (m, 4H, NCH_2_), 1.61 (s, 4H, CH_2_), 1.28 – 1.18 (m, 6H, CH_3_), 1.05 (s, 9H, CH_3_), 0.87 (s, 3H, CH_3_), 0.77 (s, 3H, CH_3_). ^13^C NMR (126 MHz, Chloroform-*d*) δ 170.79, 170.75, 170.42, 147.21, 135.61, 134.38, 134.34, 133.52, 132.75, 132.22, 131.33, 130.57, 130.28, 129.88, 129.72, 128.79, 127.84, 126.83, 116.80, 111.65, 111.44, 111.14, 62.93, 61.95, 60.54, 41.71, 41.26, 38.32, 33.68, 33.03, 30.40, 28.46, 27.94, 26.93, 19.35, 14.33, 12.43.

**Chromophore YZ5：**The blue-violet solid chromophore YZ5 was prepared by synthesizing YZ1 with a yield of 50.7%，compound 10e (0.21 g, 0.15 mmol) and the ratio of the amount of CF_3_-TCF acceptor (0.15 g, 0.49 mmol) to 1:1.5. HRMS (ESI) (M+, C_82_H_78_F_3_N_11_O_6_SSi ): calcd: 1429.5579; found: 1429.5576. ^1^H NMR (500 MHz, Chloroform-*d*) δ 7.95 (dd, *J* = 17.3, 8.2 Hz, 4H, ArH), 7.62 (d, *J* = 14.8 Hz, 1H, =CH), 7.57 (d, *J* = 7.3 Hz, 4H, ArH), 7.47 (s, 1H, =CH), 7.44 (s, 5H, ArH), 7.39 (d, *J* = 12.6 Hz, 1H, =CH), 7.34 (s, 1H), 7.32 (d, *J* = 7.2 Hz, 2H, ArH), 7.28 (d, *J* = 7.4 Hz, 4H, ArH), 7.18 (d, *J* = 8.4 Hz, 2H, ArH), 7.11 (d, *J* = 8.2 Hz, 2H, ArH), 7.00 (t, *J* = 7.2 Hz, 6H, ArH), 6.77 (d, *J* = 8.3 Hz, 2H, ArH), 6.71 (d, *J* = 11.5 Hz, 1H, =CH), 6.66 (d, *J* = 8.6 Hz, 2H, ArH), 6.35 (d, *J* = 14.3 Hz, 1H, =CH), 4.47 (dt, *J* = 22.6, 6.3 Hz, 4H, OCH_2_), 3.76 – 3.69 (m, 4H, NCH_2_), 3.66 (t, *J* = 6.9 Hz, 2H, OCH_2_), 3.53 – 3.45 (m, 4H, NCH_2_), 2.67 (t, *J* = 6.7 Hz, 2H, SCH_2_), 2.22 (s, 2H, CH_2_), 1.98 (s, 2H, CH_2_), 1.21 (t, *J* = 3.9 Hz, 6H, CH_3_), 0.99 (s, 9H, CH_3_), 0.80 (s, 3H, CH_3_), 0.73 (s, 3H, CH_3_). ^13^C NMR (126 MHz, Chloroform-*d*) δ 175.89, 165.82, 165.76, 162.03, 158.55, 155.14, 151.53, 148.41, 147.99, 147.16, 145.06, 145.01, 139.31, 135.53, 133.44, 132.72, 132.12, 131.53, 131.26, 130.68, 130.45, 130.15, 130.11, 129.92, 129.75, 128.64, 127.76, 126.97, 126.75, 126.39, 125.19, 118.97, 118.94, 116.66, 112.01, 111.66, 111.51, 111.25, 111.16, 62.85, 60.44, 48.80, 45.48, 38.26, 30.30, 29.74, 28.37, 27.83, 26.86, 19.27, 14.26, 12.45.

**Chromophore YZ6：**By synthesizing YZ1 with the same chromophore YZ1, the ratio of compound 10f (0.30 g, 0.24 mmol) to CF_3_-TCF acceptor (0.13 g, 0.41 mmol) was 1:1.5, and blue-violet solid chromophore YZ6 was prepared with a yield of 49.9 % (0.19 g, 0.12 mmol). HRMS (ESI) (M+, C_94_H_88_F_3_N_5_O_10_SSi ): calcd: 1563.5973; found: 1563.5976. ^1^H NMR (500 MHz, Chloroform-*d*) δ 8.08 (d, *J* = 22.0 Hz, 1H, =CH), 7.66 (d, *J* = 15.0 Hz, 1H, =CH), 7.62 (d, *J* = 7.4 Hz, 4H, ArH), 7.51 (d, *J* = 9.0 Hz, 2H, ArH), 7.48 (d, *J* = 4.3 Hz, 4H, ArH), 7.43 (d, *J* = 12.6 Hz, 1H, =CH), 7.37 (d, *J* = 7.3 Hz, 2H, ArH), 7.33 (d, *J* = 7.3 Hz, 4H, ArH), 7.30 (s, 3H, ArH), 7.23 (d, *J* = 8.5 Hz, 2H, ArH), 7.16 (d, *J* = 8.3 Hz, 2H, ArH), 7.05 (dd, *J* = 14.8, 11.6 Hz, 1H, =CH), 6.84 – 6.81 (m, 2H, ArH), 6.80 (d, *J* = 8.5 Hz, 2H, ArH), 6.75 (d, *J* = 11.6 Hz, 1H, =CH), 6.69 (d, *J* = 8.5 Hz, 2H, ArH), 6.38 (d, *J* = 14.4 Hz, 1H, =CH), 4.69 (d, *J* = 9.6 Hz, 8H, CH_2_), 4.51 (dt, *J* = 21.9, 6.4 Hz, 4H, OCH_2_), 3.80 – 3.73 (m, 4H, OCH_2_), 3.70 (t, *J* = 7.1 Hz, 2H, OCH_2_), 3.54 (dq, *J* = 22.9, 7.1 Hz, 4H, NCH_2_), 2.71 (t, *J* = 6.8 Hz, 2H, SCH_2_), 2.54 (s, 4H, ≡CH), 1.61 (s, 4H, CH_2_), 1.26 – 1.21 (m, 6H, CH_3_), 1.03 (s, 9H), 0.85 (s, 3H), 0.78 (s, 3H, CH_3_). ^13^C NMR (126 MHz, Chloroform-*d*) δ 166.11, 166.06, 158.69, 158.65, 158.51, 151.35, 148.33, 147.90, 147.20, 139.16, 135.60, 133.52, 132.79, 132.16, 131.98, 131.95, 131.32, 130.71, 130.62, 130.29, 130.09, 129.88, 129.71, 128.79, 127.84, 127.10, 126.82, 125.22, 116.77, 112.06, 111.66, 111.48, 111.25, 111.16, 109.15, 109.08, 107.79, 107.69, 78.01, 76.21, 76.19, 62.92, 62.60, 62.54, 57.17, 56.25, 56.23, 48.87, 48.80, 45.66, 45.58, 41.71, 41.26, 38.32, 30.38, 28.44, 27.93, 26.93, 19.35, 12.56, 12.53.

# 3. NMR pictures

**Figure S2.** ^1^H-NMR spectrum of chromophore YZ1

**Figure S3.**^13^C-NMR spectrum of chromophore YZ1

**Figure S4.** ^1^H-NMR spectrum of chromophore YZ2

**Figure S5.** ^13^C -NMR spectrum of chromophore YZ2

**Figure S6**. ^1^H -NMR spectrum of chromophore YZ3

**Figure S7.**^13^C-NMR spectrum of chromophore YZ3

**Figure S8.** ^1^H-NMR spectrum of chromophore YZ4

**Figure S9.** ^13^C-NMR spectrum of chromophore YZ4

**Figure S10.** ^1^H-NMR spectrum of chromophore YZ5

**Figure S11.** ^13^C-NMR spectrum of chromophore YZ5

**Figure S12.** ^1^H-NMR spectrum of chromophore YZ6

**Figure S13.** ^13^C-NMR spectrum of chromophore YZ6

# 4. Differential Scanning Calorimetry testing

**Figure S14**. DSC curves for crosslinking chromophores YZ1 before crosslinking

**Figure S15.** DSC curves for crosslinking chromophores YZ2 before crosslinking

**Figure S16.** DSC curves for crosslinking chromophores YZ3 before crosslinking

**Figure S17.** DSC curves for crosslinking chromophores YZ4 before crosslinking

**Figure S18**. DSC curves for crosslinking chromophores YZ5 before crosslinking

**Figure S19**. DSC curves for crosslinking chromophores YZ6 before crosslinking

**Figure S20**. DSC curves for crosslinking chromophores 2:1 YZ1/YZ2 before crosslinking

**Figure S21.** DSC curves for crosslinking chromophores 2:1 YZ1/YZ2 after crosslinking

**Figure S22.** DSC curves for crosslinking chromophores 1:1 YZ1/YZ3 before crosslinking

**Figure S23**. DSC curves for crosslinking chromophores 1:1 YZ1/YZ3 after crosslinking

**Figure S24**. DSC curves for crosslinking chromophores 1:1 YZ3/YZ4 before crosslinking

**Figure S25**. DSC curves for crosslinking chromophores 1:1 YZ3/YZ4 after crosslinking

**Figure S26.** DSC curves for crosslinking chromophores 2:1 YZ5/YZ6 before crosslinking

**Figure S27.** DSC curves for crosslinking chromophores 2:1 YZ5/YZ6 after crosslinking

# **5. UV-Vis Absorption Spectroscopy**

In this study, the UV absorption of chromophore YZ1-YZ6 in seven solvents with different polarities was tested in Acetonitrile, Acetone, 1,4-Dioxane, Tetraphydrofuran, Dichloromethane, Chloroform and Methylbenzene, as shown in Figure S28. Due to the same body, the chromophore YZ1-YZ6 all showed obvious main peaks at the absorption wavelength from 600 nm to 1200 nm. In addition, under the solvent test of acetone, acetonitrile and dichloromethane, YZ1-YZ6 showed a more obvious shoulder peak at about 1100 nm, considering that the dielectric environment of these three solvents was much higher than that of the molecule itself, the solvent molecule was strongly polarized, and the intramolecular charge transition (ICT) energy level splitting of the chromophore was induced, that is, the polar solvent made the charge separation between the donor and the acceptor more significant, resulting in the split of the original single ICT transition into multiple excited states^[2],^ which was spectrally manifested as a shoulder near 1100 nm. As shown in Figure S28 [a], the functionalized anthracene contained in YZ1 exhibits an obvious "mountain-shaped" absorption peak from 300 nm to 400 nm^[3],^ which verifies the correctness of the molecular structure.

**Figure S28.** [a] [b] [c] [d] [e] [f] are chromophore YZ1-YZ6 respectively put in seven aprotic solvents with different dielectric constants of Normalized UV-Vis absorption spectra.

# 6.Electro-optical performance test

The electro-optical coefficient r_33_ was tested on the YZ1-YZ6 chromophore and its mixed cross-linking products by the Teng-Man test method, and the effect of cross-linking reaction of different functionalized groups on the electro-optical properties of the chromophore was further explored. The test curves of the polarized electric field and r_33_ of the individual chromophore YZ1-YZ6 obtained are shown in Figure S29.

**Figure S29.** Polarization plot of r_33_ and polarization field before and after crosslinking of individual chromophore

# 7 Properties of the state-of-the-art organic EO materials

**Figure S30**.The chemical structure of the state-of-the-art organic EO material and the molecular structure of the YZ series.

**Table S1** Properties of the state-of-the-art organic EO materials

| **Cmpd** | **T_d_ (°C)** | **T_g_ (°C)** | **β_tot_^a^ (10^-30^esu)** | **max. r_33_/(pm/V)** |
| --- | --- | --- | --- | --- |
| **BAY1** | 205 | 84 | 2941 | 460 or 1100 （with TiO_2_） |
| **M1a-FP-ON** | 233 | -- | 376 | 127 |
| **P1** | -- | 172 | -- | 223 |
| **2：1 YZ1/YZ2** | 254/291 | 187 | 1284/1387 | 289 |
| ^a^ was the first-order hyperpolarizability in vacuum calculated from DFT calculations. | | | | |

The best-performing YZ1/YZ2 crosslinking system in the YZ series is compared to state-of-the-art organic materials, including single chromophore (BAY1) [4] , guest-host system (M1a FP-ON) [5], and polymer (P1) [6] . As can be seen from Table S1, the thermal stability of 2:1 YZ1/YZ2 is better than that of the former, but the first-order hyperpolarizability and electro-optic coefficient exhibited are lower than BAY1. On the whole, the YZ series is still a good chromophore cross-linking structure, which solves the problem of low stability in the front, and the performance of r_33_ can still reach close to 300 pm/V, which is one of the organic optical materials with the best comprehensive performance.

# 8. Comparison of different systems

**Figure S31.** The chemical structure of BLD1 and BLD3 is related to the molecular structure of YZ1/YZ2 and its cross-linking mode.

**Table S2** Properties of the state-of-the-art organic EO materials

| **Cmpd** | **T_d_ (°C)** | **T_g_ (°C)** | **max. r_33_/(pm/V)** |
| --- | --- | --- | --- |
| **BLD1** | 207 | 70 | 281 |
| **BLD3** | 225 | 66 | 351 |
| **2：1 YZ1/YZ2** | 254/291 | 187 | 289 |

BLD1 and BLD3 designed by Chen[1] et al. are shown in Figure S31. In 4,4'-bis(diethylamino)benzophenone, aniline (diethylamino) electron-donor groups are connected to both sides of the carbonyl group, which can effectively enhance the electron-donating ability of the molecule. Among them, the r_33_ value of BLD3 is 351 pm/V, which is 25 % higher than that of BLD1 (281 pm/V). The T_d_ of the BLD series is above 200 °C and shows good thermal stability, but the glass transition temperature T_g_ is below 75 °C, which limits its long-term use in practical devices. Therefore, the YZ series designed for binary cross-linking, still taking 2:1 YZ1/YZ2 as an example, the cross-linked T_g_ is more than double that of the BLD series, and the T_d_ is above 250 °C, showing excellent thermal stability performance, and the electro-optical performance is still in a high state, which proves the necessity of YZ design.

# 9. Reference

[1] Q.Z. Zeng, X.Y. Chen, A. Rahman, Z.Y. Zeng, Z.W. Liang, L. Shi, Z.L. Huang, S.H. Bo, F.G. Liu, J.H. Wang, A modifiable double donor based on bis(N-ethyl-N-hydroxyethyl)aniline for organic optical nonlinear chromophores, Materials Chemistry Frontiers, 6 (2022) 1079-1090.

[2] A.K. Jen, G.W. Lu, Charge Transfer in D-π-A Structured Chromophores: A Spectroscopic Study, Journal of Applied Physics, 78 (1995) 567-578.

[3] S.R. Marder, J.W. Perry, Design of Organic Materials with Large Second-Order Optical Nonlinearities, Journal of the American Chemical Society, 102 (1980) 7520-7522.

[4] H.J. Xu, D.L. Elder, L.E. Johnson, Y. de Coene, S.R. Hammond, W. Vander Ghinst, K. Clays, L.R. Dalton, B.H. Robinson, Electro-Optic Activity in Excess of 1000 pm V^-1^ Achieved via Theory-Guided Organic Chromophore Design, Advanced Materials, 33 (2021) 2104174.

[5] D. Zhang, J. Zou, W.L. Chen, S.M. Yiu, M.K. Tse, J.D. Luo, A.K.Y. Jen, Efficient, Stable, and Scalable Push-Pull Heptamethines for Electro-Optics, Chemistry of Materials, 34 (2022) 3683-3693.

[6] G.-W. Lu, J. Hong, F. Qiu, A.M. Spring, T. Kashino, J. Oshima, M.-a. Ozawa, H. Nawata, S. Yokoyama, High-temperature-resistant silicon-polymer hybrid modulator operating at up to 200 Gbit s−1 for energy-efficient datacentres and harsh-environment applications, Nature Communications, 11 (2020) 4224.
